# Accelerating drug development for amyotrophic lateral sclerosis: construction and application of a disease course model using historical placebo group data

**DOI:** 10.1186/s13023-024-03057-5

**Published:** 2024-02-02

**Authors:** Ruifen Cai, Juan Yang, Lijuan Wu, Yixiao Liu, Xinrui Wang, Qingshan Zheng, Lujin Li

**Affiliations:** 1 Center for Drug Clinical Research, Shanghai University of Traditional Chinese Medicine, No.1200 Cailun Road, Shanghai, 201203 China; 2 State Key Laboratory of Integration and Innovation of Classic Formula and Modern Chinese Medicine, Shanghai University of Traditional Chinese Medicine, Shanghai, China

**Keywords:** Amyotrophic lateral sclerosis, Disease course, Placebo, Model-based meta-analysis (MBMA)

## Abstract

**Background:**

Amyotrophic lateral sclerosis (ALS) is an irreversible degenerative disease. Placebo-controlled randomized trials are currently the main trial design to assess the clinical efficacy of drugs for ALS treatment. The aim of this study was to establish models to quantitatively describe the course of ALS, explore influencing factors, and provide the necessary information for ALS drug development.

**Methods:**

We conducted a comprehensive search of PubMed and the Cochrane Library Central Register for placebo-controlled trials that evaluated treatments for ALS. From these trials, we extracted the clinical and demographic characteristics of participants in the placebo group, as well as outcome data, which encompassed overall survival (OS) and Amyotrophic Lateral Sclerosis Functional Rating Scale (ALSFRS-R) scores, at various time points.

**Results:**

In total, 47 studies involving 6118 participants were included. Disease duration and the proportion of patients receiving riluzole were identified as significant factors influencing OS in the placebo group. Specifically, the median OS was 35.5 months for a disease duration of 9 months, whereas it was 20.0 months for a disease duration of 36 months. Furthermore, for every 10% increase in the proportion of patients treated with riluzole (100 mg daily), there was an association with a median OS extension of approximately 0.4 months. The estimated time for the ALSFRS-R score in the placebo group to decrease to 50% of its maximum effect from baseline level was approximately 17.5 months, and the time to reach a plateau was about 40 months.

**Conclusions:**

The established disease course model of the historical placebo group is valuable in the decision-making process for the clinical development of ALS drugs. It serves not only as an external control to evaluate the efficacy of the tested drug in single-arm trials but also as prior information that aids in accurately estimating the posterior distribution of the disease course in the placebo group during small-sample clinical trials.

**Supplementary Information:**

The online version contains supplementary material available at 10.1186/s13023-024-03057-5.

## Introduction

Amyotrophic lateral sclerosis (ALS), an irreversible degenerative disease involving the upper and lower motor neurons [1], is accompanied by clinical manifestations, such as progressive muscle atrophy, progressive limb paralysis, and eventual death due to respiratory failure. The median survival time for patients with ALS is 3–5 years [2, 3], with a prevalence of 4–6 per 100,000; the incidence of ALS is higher in males than in females [4]. ALS can be attributed to a combination of genetic, epigenetic, and environmental factors; however, its pathogenesis remains unknown [5]. Currently, Food and Drug Administration (FDA)-approved drugs for ALS include riluzole, edaravone, and AMX0035. Riluzole, an anti-glutamate drug, exerts its pharmacological effects by inhibiting the release of neurotransmitters, such as glutamate and aspartate, in the brain. Its neuroprotective properties involve suppressing the activity of excitatory amino acids and stabilizing the inactivated state of voltage-dependent sodium channels. However, the observed survival extension attributed to riluzole, as reported in a meta-analysis, is only 3 months [6]. Edaravone is a free-radical scavenger that provides neuroprotective support in the nervous system and potentially delays disease progression in patients with ALS. It was approved in 2017; however, its clinical efficacy remains controversial [7, 8]. AMX0035 is a fixed-dose combination of tauroursodeoxycholic acid and sodium phenylbutyrate. Although its mechanism of action remains poorly understood, the two compounds function together to prevent nerve cell death, possibly by blocking stress signals within the mitochondria and endoplasmic reticulum. After the completion of a phase II trial [9], Relyvrio was conditionally approved by the FDA in September 2022, although its final efficacy needs to be confirmed in a phase III trial.

Because the clinical therapeutic endpoints for ALS remain largely unmet, safe and effective drugs to treat ALS are needed. Placebo-controlled randomized trials are currently the main trial designs for assessing the clinical efficacy of drugs for the treatment of ALS. However, as ALS is a rare disease, the limited clinical resources hinder investigations into the clinical efficacy of the growing number of new drugs. During early clinical trial stages, the historical placebo group in the ALS clinical trial can be used as an external control group. Effective drugs with potential development value are then selected for confirmatory clinical trials. In this way, the hurdle of the shortage of clinical resources can be overcome, costs can be reduced, and patient participation in new drug trials can be attracted. However, given the heterogeneity between trials, a large difference may exist between the historical placebo group and the trial, hindering the accurate evaluation of the efficacy of the test drug.

A model-based meta-analysis (MBMA) combines pharmacometric modeling methods with meta-analysis. Unlike classical meta-analysis, an MBMA can be used to summarize and analyze data from each visit time point by constructing a time-course model, enabling the evaluation of the entire time-effect process. An MBMA also addresses heterogeneity among studies by implementing a covariate model, mitigating biases resulting from factors such as patient characteristics and study design [10, 11]. Building on the aforementioned model, an MBMA can quantify the time course and influential factors of the placebo response. Therefore, this is an ideal strategy for constructing accurate external controls. Based on accumulated evidence, in the present study, overall survival (OS) and the Amyotrophic Lateral Sclerosis Functional Rating Scale (ALSFRS-R) were used as the primary indicators to establish a disease course model for the historical placebo group in ALS clinical trials. We used an MBMA to provide an accurate external control for drug efficacy judgment and decision-making in early ALS clinical trials.

## Methods

### Search strategy

We searched the PubMed and Cochrane Library databases for placebo-controlled randomized clinical trials assessing ALS treatment, with a search deadline of February 21, 2022. The literature was limited to clinical trials and the language was limited to English. The specific search strategy is described in Additional file 1: Method 1 and Additional file 1: Table S1. Relevant reviews and websites were manually examined to identify additional studies that may have been overlooked.

### Inclusion criteria

The inclusion criteria were as follows: (1) placebo-controlled randomized trials of drugs with a sample size greater than 10; (2) the participants in this study were adult patients who met diagnostic criteria such as the El Escorial criteria for a definitive or probable diagnosis of ALS; (3) studies had a crossover design, with data extracted only for the first treatment period; and (4) the endpoints were OS or ALSFRS-R scores at different time points. There are two versions of the ALSFRS-R; to avoid heterogeneity, we only included studies using the ALSFRS-R version approved in 1999 (0–48 points).

As stated in the FDA drug development guidelines, OS and ALSFRS-R, which were revised in 1999, are important endpoints for clinical trials [12, 13]. OS was defined as the duration from the date of enrollment in the trial to the occurrence of death, tracheostomy, or the initiation of continuous ventilatory support. The ALSFRS-R (0–48) rating scale assesses the rate of disease progression based on four major domains: bulbar function (swallowing and speech), fine motor function, gross motor function, and respiratory function. The score is deemed proportional to patient function [14, 15].

### Data extraction

A database was created using Microsoft Excel 2010 and the following information was entered for the included studies: literature characteristics (authors, year of publication, clinical trial registration number, and region), trial design (test drug, sample size, and treatment duration), clinical outcomes (OS and mean change from baseline ALSFRS-R score at each time point in the placebo group), and subject characteristics. The subject characteristics included in the analysis were as follows: age at onset (referring to the age of the patient at the onset of the initial symptom), proportion of male participants, disease duration, proportion of individuals with bulbar onset, baseline ALSFRS-R score, administration of basic treatment (i.e., medications such as vitamins and coenzyme Q10, which have shown no positive effect in clinical trials for ALS), and proportion of patients receiving riluzole treatment.

Engauge Digitizer (version 2.25.0.32) was used for data extraction from the graphics. Two investigators independently extracted all information, and inconsistencies were resolved by consulting with a third investigator.

### Risk of bias assessment

The risk of bias for the included studies was evaluated using the Cochrane Risk of Bias Tool [16], which includes random sequence generation, allocation concealment, blinding of participants and personnel, blinding of outcome assessment, incomplete outcome data, selective reporting, and other bias. The term “other bias” was defined as trial sponsorship by drug companies and an incomparable baseline for the subjects in each trial group. Each item is graded as low, high, or unclear. Two investigators independently scored the quality of the literature and a third investigator adjudicated any discrepancies.

### Modeling analysis of OS and ALSFRS-R

A parametric survival model was used to analyze the OS data, and the Sigmoid Emax model was employed to describe the mean change in the ALSFRS-R score from baseline. Model building included the establishment of structural effects, random effects, and covariate models. A detailed description of this process is provided in Additional file 1: Method 2.

After constructing the final model, model diagnostic plots were used to evaluate the goodness-of-fit of the model [17], and the predictive performance of the model was assessed by comparing the model predictions with observations using a Visual Predictive Check (VPC) [18]. The robustness of the model was assessed using a nonparametric bootstrap with 1000 repetitions of the final model [19]. The bootstrap median and 95% confidence intervals (CIs) of the parameter values were compared with those estimated from the final model.

### Typical value simulation and subgroup analyses

Based on model parameter estimations, the typical values and 95% confidence intervals (CIs) of the placebo group at different covariate levels were simulated using 1000 Monte Carlo simulations. Furthermore, a subgroup analysis was performed to investigate potential influential factors that were of particular interest irrespective of their inclusion in the covariate model construction. Factors considered in the subgroup analysis included age at onset, proportion of male participants, percentage of patients treated with riluzole, proportion of individuals with bulbar onset, disease duration, baseline ALSFRS-R score, publication year, and administration of basic treatment. The analytical method consisted of two steps. First, individual parameters and standard errors were obtained for each study after eliminating covariate effect differences using Bayesian post-hoc estimation. Second, a meta-analysis with a random-effects model was used to summarize the typical values of the pharmacodynamic parameters and their standard errors for each predefined subgroup. Based on the results, typical values and 95% CIs for each subgroup were simulated using 1000 Monte Carlo simulations.

### Model application

One potential application of the final model is the assessment of drug efficacy in single-arm clinical trials of ALS. We identified two single-arm ALS trials [20, 21], each of which reported the endpoints we required (a single-arm clinical trial with reported endpoints that we used for modeling was sufficient). One trial reported the OS for 87 subjects after lithium carbonate treatment and the other reported the ALSFRS-R scores of 18 subjects after receiving VM202 treatment. The specific method was based on the final model to simulate the response of the placebo group, with characteristics similar to those in the single-arm trial. If the efficacy observed in the trial was better and exceeded the 95% CI of the historical placebo group, it would suggest that the efficacy of the test drug was significantly better than that of the placebo, and that the test drug has the potential for further development.

Another potential application involves utilizing the disease course of the historical placebo group as a priori information to estimate the posterior distribution of the disease course within the placebo group in placebo-controlled randomized trials with limited sample sizes, particularly when evaluating the efficacy of a test drug. This approach aims to enhance the accuracy of disease progression estimation in the placebo group, offering valuable insights for clinical trials with constrained participant numbers. A phase II clinical trial was conducted to investigate the initial efficacy of talampanel, an orally active, noncompetitive antagonist of alpha-amino-3-hydroxy-5-methyl-4-isoxazolepropionic acid, as a potential treatment for ALS. Although the study had a limited sample size, the research team expressed a strong desire to conduct a larger clinical trial to validate these results. Our objective was to explore the possibility of predicting trial outcomes without the need for additional clinical studies. In total, 59 subjects were enrolled in the trial, of whom 40 received talampanel and 19 received a placebo. Compared with baseline values, ALSFRS-R at 9 months decreased by 7.1 and 10.1 points in the talampanel and placebo groups, respectively [22]. Accordingly, talampanel-treated patients declined 30% less quickly than placebo-treated patients, considering the ALSFRS-R values, which were well within the clinically significant range for physicians specializing in ALS. However, a large sampling error may have occurred owing to the small sample size of the placebo group. To accurately assess the efficacy advantage of talampanel, we considered the response of the historical placebo group a priori to estimate the posterior distribution of the placebo group in this trial, which was based on the final model. The placebo intervals for the large sample condition were simulated, and the posterior estimation intervals were obtained through Bayesian feedback [23].

### Software

Model establishment and simulations were performed using NONMEM 7.4 (ICON Development Solutions, USA). Model parameters were estimated using a first-order conditional estimation method with interaction. Statistical analysis and plot generation were performed using R 4.0.3 (The R Foundation of Statistical Computing). Bayesian posterior estimation was performed using WinBUGS. Finally, a literature quality assessment was performed using Review Manager 5.4 (Cochrane Collaboration, London, UK).

## Results

### Characteristics of included studies

**Fig. 1:**
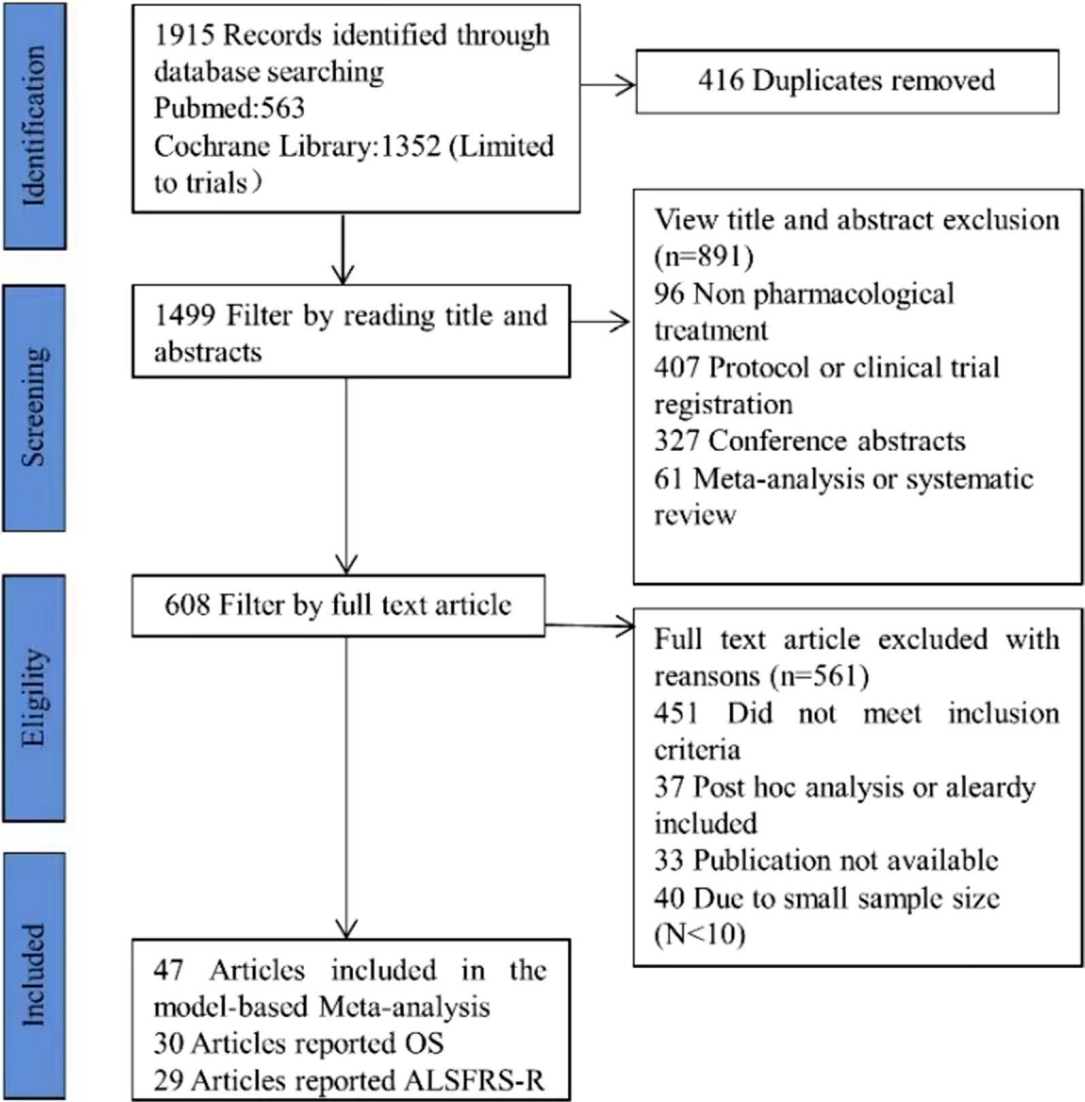
Flow chart of literature screening

In total, 47 studies involving 6118 subjects were included in the analysis (Additional file 1: Table S2). Figure 1 shows the detailed literature screening process. 30 studies (N = 3781) reported OS data and 29 (N = 2337) reported ALSFRS-R scores. The sample size of the placebo group ranged from 11 to 468 subjects (median, 75), and the mean baseline ALSFRS-R score ranged from 30.74 to 43.24 (median, 38.1). The baseline characteristics of the included studies are summarized in Table 1, and more details are shown in Additional file 1: Table S3. Among the 47 selected studies, the overall quality was assessed as high, and approximately 48.9% of the included trials were judged to have a low risk of bias. Detailed information regarding the quality assessment of the literature is provided in Additional file 1: Figure S1.

**Table 1:** Brief characteristics of the included studies: median (minimum–maximum)

	OS	ALSFRS-R	ALL
Number of arms	30	29	59
Sample size per arm	100 (11–468)	52 (11–441)	75 (11–468)
Age, year	56.2 (53.5–64.6)	56.0 (51.2–60.5)	56.1 (51.2–64.6)
Gender, male%	64 (45.5–71)	61.3 (45.5–72.0)	61.3 (45.5–72)
Disease duration, month	17.8 (9–36)	17.5 (9.6–26.4)	17.6 (9–36)
Riluzole therapy, %	90.95 (0–100)	96.45 (50–100)	87.75 (0–100)
ALSFRS-R score at baseline	38.1 (30.74–42)	38.15 (32.6–43.24)	38.1 (30.74–43.24)

### Model establishment and evaluation

The log-normal hazard function model was selected as the base hazard function model to describe the changes in OS over time. Covariate analysis revealed that disease duration and the percentage of riluzole-treated patients significantly affected the hazard function. The final model parameters are presented in Table 2. The covariate model is expressed as follows:

**Table 2:** Parameter estimation of the final mode of OS

	Estimate	RSE (%)	Bootstrap (983)^*^ Median (95% CI)
SIGM	0.916	5.80	0.920 (0.820–1.03)
MU	3.04	2.80	3.06 (2.87–3.26)
θ_Duration_	0.0317	28.3	0.0311 (0.0109–0.0511)
θ_Riluzole_	− 0.228	57.5	− 0.217 (− 0.508–0.101)
η (SIGM), %	25.2	15.6	23.8 (16.2–31.5)
η (MU), %	7.20	19.4	6.70 (4.00–10.1)
ε_add_	0.693	17.2	0.686 (0.503–0.918)

*SIGM* the median of the lognormal distribution, *MU* the variance of the lognormal distribution, *θ*_*Duration*_ is the covariant parameter of the disease duration, *θ*_*Riluzole*_ is the is the covariant parameter of the proportion of patients treated with Riluzole, *η* is the inter-study variability of model parameter, *ε*_*add*_ additive residual error; RSE, relative standard error, *CI* confidience interval

^*^983 means that the model succeeded 983 times out of 1000 bootstrap attempt

1 $${\text{h}}\left({\text{t}}\right)=h(t){\_}_{Base}*{e}^{\left({\text{DURATION}}-17.8\right)*0.0317-{\text{RILUZOLE}}*0.228}$$

In Eq. 1, h(t)__Base_ is the base hazard function, DURATION indicates the time from first symptom onset to enrollment, 17.8 is the median duration of the patients, RILUZOLE indicates the percentage of patients receiving riluzole treatment, and 0.0317 and -0.228 are the covariate coefficients of the hazard function.

The base model was deemed the final model as no covariables related to the ALSFRS-R model parameters were detected (Additional file 1: Figure S2).

**Fig. 2:**
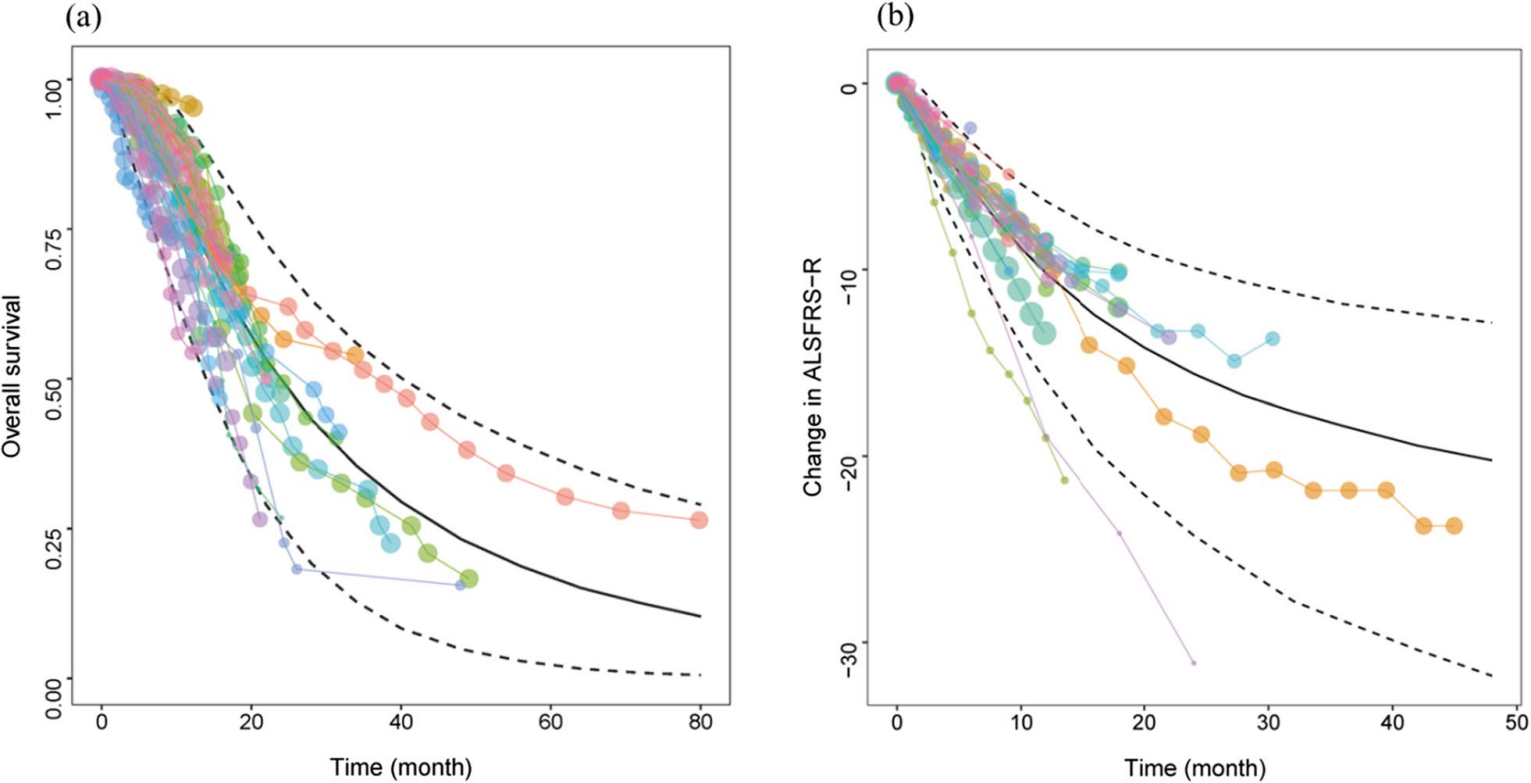
Visual prediction check (VPC) of the final model of OS (**a**) and ALSFRS-R (**b**). Note: Points represent the observed OS and ALSFRS-R data, with point size proportional to the number of patients in each arm; The solid line is the model-predicted median of the disease course, and the dotted lines are the model-predicted 2.5th and 97.5th percentiles of the disease course, respectively

The model diagnostic plots showed that the observations (OBS), population predictions (PRED), and individual predictions (IPRED) of the final OS and ALSFRS-R models were evenly distributed on both sides of the diagonal line, with fitted lines coinciding with the diagonal lines. The conditional weighted residual errors (CWRES) of most points were distributed evenly around the 0 line within 6, and the fitting lines of CWRES *vs*. PRED and CWRES *vs*. time nearly coincided with the 0 line. Thus, the model had a good fit with the observed values without any notable bias (Additional file 1: Figure S3). One-thousand repeated bootstraps were performed on the OS and ALSFRS-R models, and 983 (Table 2) and 999 (Table 3) iterations, respectively, were successful. The parameter estimation values generated by bootstrapping approximated those of the original dataset, indicating the robustness of the model. The VPC plot revealed that most observed data fell within the model-predicted 95% CI (Fig. 2), indicating that the model had a good predictive performance.

**Table 3:** Parameter estimation of the final model of ALSFRS-R

	Estimate	RSE (%)	Bootstrap (999)^*^ Median (95% CI)
E_max_ (Score)	26.2	7.0	27.3(23.4–46.4)
ET_50_ (month)	17.5	4.4	17.7(16.6–38.8)
γ	1.2	5.8	1.18(1.01–1.29)
η (E_max_), %	25.8	18.0	25.7(12.8–31.9)
η (γ), %	12.2	20.0	11.9(7.7–17.3)
ε_add_	4.00	14.9	3.88 (2.61–5.10)

*E*_*max*_ the maximum effect value, *ET*_*50*_ time to reach half of the maximum effect value, *γ* the shape parameter, *η* is the inter-study variability of model parameter, *ε*_*add*_ additive residual error, *RSE* relative standard error, *CI* confidience interval

^*^999 means that the model succeeded 999 times out of 1000 bootstrap attempt

### Disease course of historical placebo group

**Fig. 3:**
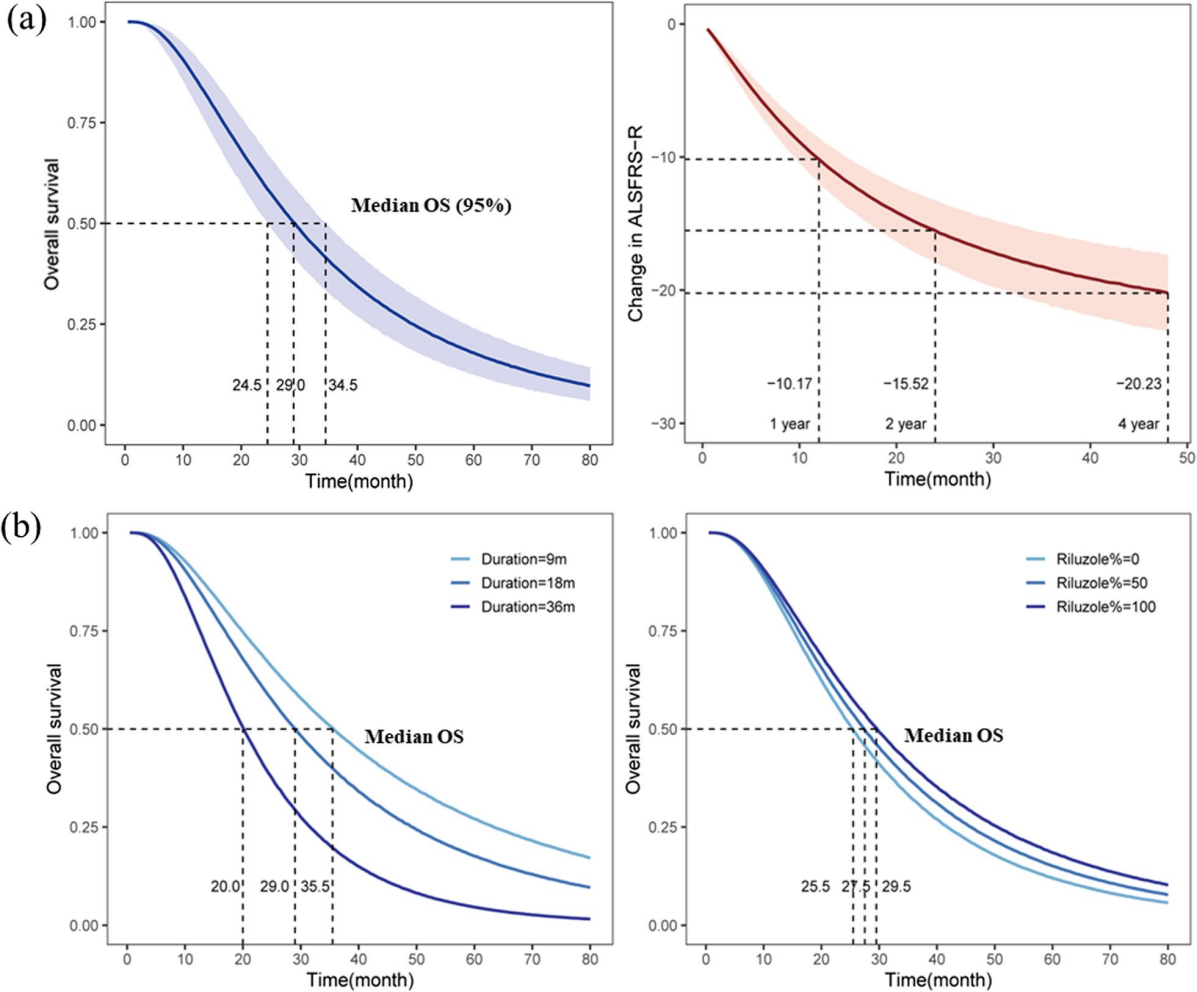
**a** Typical value and 95%CI of placebo group in clinical trials of ALS (OS and ALSFRS-R); **b** The typical OS under different baseline levels. Note: **a** The typical median OS (that is, the median duration of the patients was 17.8 months, and 90% of patients received Riluzole treatment) of placebo group was 29.0 (95% CI 24.5–34.5) months; and after treatment for 1, 2, and 4 years, the typical change from baseline ALSFRS-R scores in the placebo group were − 10.17 (95% CI − 11.92, − 8.55), − 15.52 (95% CI − 17.86, − 13.21), and − 20.23 (95% CI − 23.15, − 17.31), respectively. **b** the proportion of patients using Riluzole was fixed at 90%, when the disease duration was 9, 18 and 36 months, the median OS was 20.0, 29.0 and 35.5 months, respectively; the disease duration was fixed at 17.8 months, when the proportion of patients using Riluzole was 0%, 50% and 100%, the median OS was 25.5, 27.5 and 29.5 months, respectively

Based on the final model, the typical value distributions of OS and ALSFRS-R in the placebo group were simulated. When the median duration of the patients was 17.8 months, and 90% of patients received riluzole treatment, the typical median OS of the placebo group was 29 (95% CI 24.5–34.5) months. The 1-, 2-, and 5-year OS rates were 86% (95% CI 80–92%), 60% (95% CI 51–68%), and 18% (95% CI 12–24%), respectively (Fig. 3a).

Disease duration and percentage of patients treated with riluzole significantly affected OS. Considering disease durations of 9 and 36 months (Disease duration in the patients included in the study: 9–36 months), the median OS was 35.5 and 20.0 months, respectively. The 1-year OS rates were 89% and 77%, 2-year OS rates were 68% and 40%, and 5-year OS rates were 27% and 5%, respectively. Considering 0% and 100% of patients treated with riluzole, the median OS was 25.5 and 29.5 months, respectively. The 1-year OS rates were 83% and 87%, 2-year OS rates were 53% and 60%, and 5-year OS rates were 12% and 18%, respectively (Fig. 3b).

After treatment for 1, 2, and 4 years (maximum observation duration was 48 months), the typical change from baseline ALSFRS-R scores in the placebo group was − 10.17 (95% CI − 11.92, − 8.55), − 15.52 (95% CI − 17.86, − 13.21), and − 20.23 (95% CI − 23.15, − 17.31), respectively (Fig. 3a).

### Subgroup analysis

**Fig. 4:**
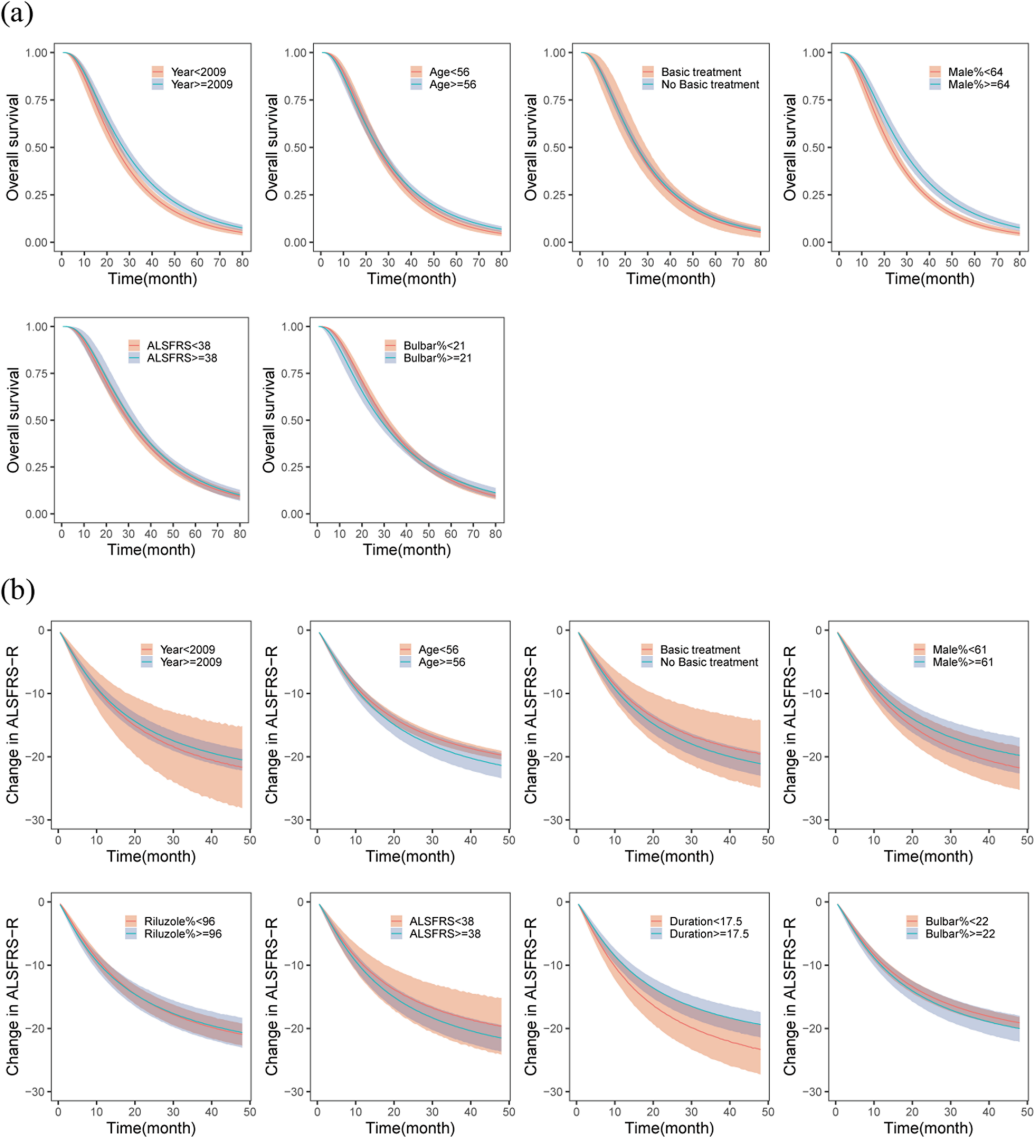
The results of subgroup analyses of OS (**a**) and ALSFRS-R (**b**). Note: In the subgroup analysis, the cut-off value for a continuous variable is determined as the median of that variable. Regarding the year of publication, 2009 serves as a cut-off value because the American Academy of Neurology Quality Standards Subcommittee updated their guidelines in that year, highlighting the importance of multidisciplinary care in optimizing medical services and prolonging survival in patients with ALS. Year, the time of publication; Age, age at onset of disease; Basic treatment, whether to receive symptomatic or other treatment; Male, the percentage of men; Riluzole, Proportion of patients receiving riluzole; ALSFRS-R, the Baseline ALSFRS-R scores valve; Duration, the time since first symptom onset; Bulbar, proportion of individuals with bulbar onset

After correcting for disease duration and percentage of riluzole-treated patients to the median level, subgroup analysis revealed that the proportion of males and year of publication were related to OS. The median OS of the subgroup with a high male proportion (> 64%) increased by 4.5 months compared with that of the subgroup with a low male proportion (< 64%). Furthermore, the median OS in studies published after 2009 increased by 3 months compared with that in studies published before 2009. Age, the proportion of patients with bulbar onset, baseline ALSFRS-R score and whether basic treatment was administered did not significantly affect OS (Fig. 4a).

Age, proportion of males, percentage of riluzole-treated patients, proportion of individuals with bulbar onset, disease duration, baseline ALSFRS-R score, publication year, and whether basic treatment was administered were not significantly correlated with the ALSFRS-R score (Fig. 4b).

### Model application

**Fig. 5:**
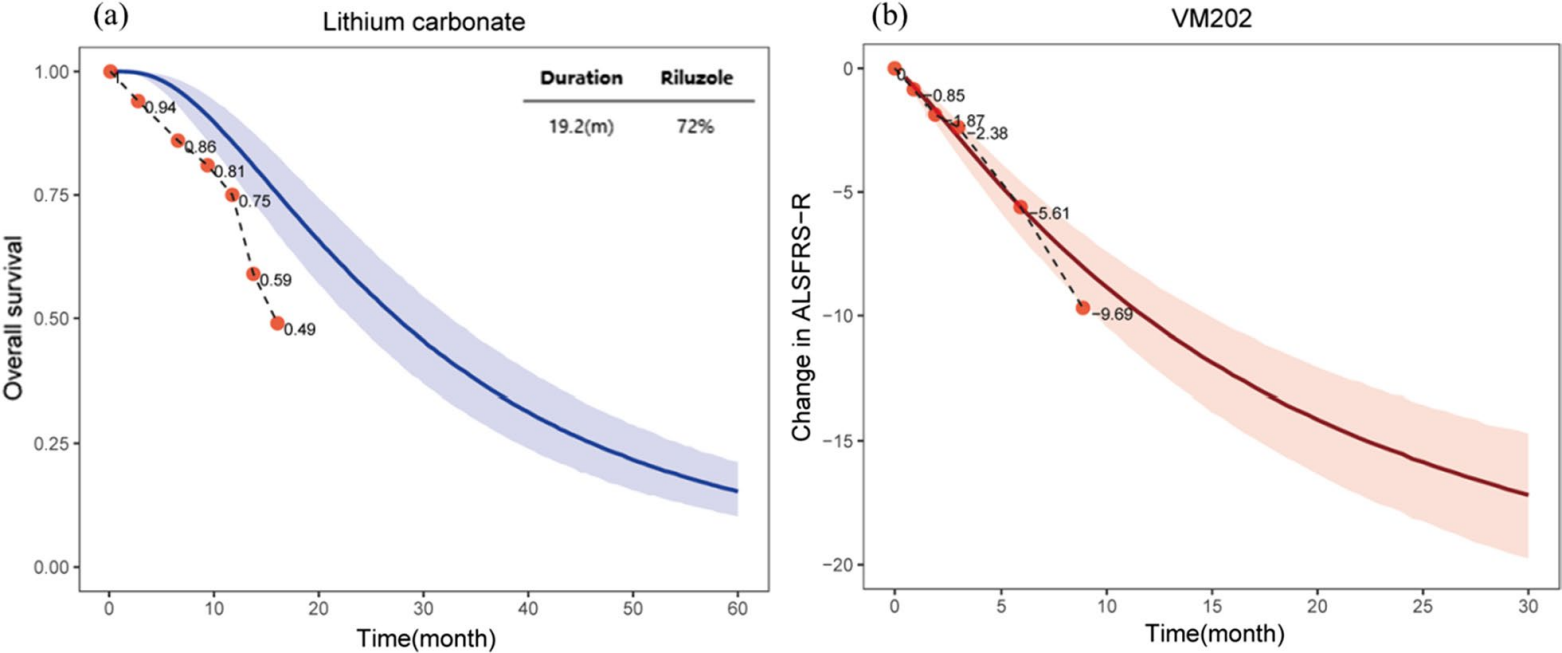
Comparison between the efficacy of the test drug in single-arm trials and that of the virtual placebo group under the same conditions. Note: **a** Overall Survival of Lithium carbonate; **b** ALSFRS-R of VM202; The black dashed line epresents the typical value of the placebo group at the same baseline predicted by the model, the shading represents its 95% CI, and the dots represent the measured efficacy in the single-arm trial; m, month

Using a historical placebo group as an external control, we evaluated the efficacy of drugs in two single-arm ALS clinical trials [20, 21]. The disease duration and percentage of patients treated with riluzole in a single-arm trial of lithium carbonate were 19.2 months and 72%, respectively. The matching OS distribution in the placebo group under the same conditions was simulated using the OS model. Based on the results, the OS within 16 months of lithium carbonate administration was lower than the 95% CI of the typical value in the historical placebo group (Fig. 5a), suggesting that lithium carbonate failed to effectively prolong the OS of patients with ALS. The changes in ALSFRS-R scores compared with baseline in another single-arm trial assessing VM202 were within the 95% CI of the typical placebo response (Fig. 5b), suggesting that there was no significant difference in ALSFRS-R score improvements between the VM202 and placebo groups.

This study incorporated the disease course of the historical placebo group as a priori information to precisely estimate the posterior distribution of the disease course within the placebo group with a small sample size. In a phase II clinical study, the change in ALSFRS-R score from baseline was − 10.1 (95% CI − 12.75, − 7.45) in the placebo group [22]. We simulated the typical value of the historical placebo group at 9 months based on the updated model, which was reconstructed after deducting the data of this test trial, and the score was − 8.07 (95% CI − 9.57, − 6.72). Utilizing this as a priori information, the posterior distribution of the placebo group in the trial was estimated to be − 8.53 (95% CI − 9.79, − 7.27), which closely resembled the efficacy observed in the drug group in this trial (− 7.1, 95% CI − 9.04, − 5.16) [23]. These results suggest that the ALSFRS-R score reduction induced by talampanel was 17% lower than that attributed to placebo. Consequently, talampanel was deemed to have a low probability of success in phase III clinical trials.

## Discussion

To date, only riluzole has been confirmed to effectively prolong the survival of patients with ALS by approximately 3 months [24]. There is a substantial unmet clinical need in the treatment of ALS, warranting the urgent development of novel and effective drugs. Placebo-controlled randomized trials are the gold standard for confirming drug effectiveness. The ALS Clinical Trial Guidelines and Community Guidance emphasize that randomized controlled trials remain the most robust method to demonstrate efficacy [13]. However, given the irreversible course of ALS, most patients are paralyzed and die within 3–5 years. Owing to ethical factors, a placebo group without intervention will fail to pass the ethical review; thus, most trials allow patients to use clinically approved drugs as background treatment. Considering the trial included in the present study, 0–100% of patients in the placebo group received riluzole as background treatment, with a median of approximately 90%. As differences in the proportions of riluzole-treated patients may affect the disease course of the placebo group, quantifying factors that impact the disease course of the placebo group in ALS clinical trials could help accurately estimate the sample size in future clinical trials and precisely determine drug efficacy in single-arm trials.

The present study quantitatively analyzed the disease course of the placebo group and factors affecting OS and ALSFRS-R in ALS clinical trials. Unlike previous studies that relied on data analysis from the Pooled Resource Open Access ALS Clinical Trials (PROACT) database [25], this study extensively incorporated a broader range of literature data. Furthermore, in contrast to the European Network for the Cure of ALS survival (ENCALS) model [26], the disease course model developed in this study was specifically designed to predict disease progression within the placebo group in ALS clinical trials rather than for individual patients. In summary, this study offers a comprehensive exploration of the characteristics associated with the disease course in a placebo group, providing valuable insights from various perspectives. This also serves as a valuable complement to the current methodology that utilizes individual-level data from the PROACT database as an external control [27–31].

In this study, the percentage of riluzole-treated patients significantly affected OS. For every 10% increase in the proportion of patients treated with riluzole, the median OS in the placebo group increased by approximately 0.4 months. One study reported that age can negatively affect OS, with a 3% risk of early death increasing every additional year [32]. However, we failed to detect any significant effect of age on OS. The present study was based on summary data from the available literature; the average age of included subjects ranged between 51.19 and 64.6 years (age of onset: 21.0–52.50). Owing to the narrow age distribution, the influence of age could not be examined effectively. Similarly, previous studies [33, 34] have suggested that the site of onset and baseline ALSFRS-R values are significant factors in the progression of ALS. However, due to a high rate of missing data for the site of onset and baseline ALSFRS-R values reported in the literature, and because our study involves aggregated-level data, the detection power for identifying influential factors was limited. Consequently, our study did not observe a significant impact of these two factors on OS and ALSFRS-R outcomes. In addition to the covariate analysis, a subgroup analysis of the variables of interest was performed. Although ALS is more common in males, the subgroup analysis revealed that the median OS of male subjects was significantly longer than that of females, consistent with the findings of a previous study [35]. Furthermore, a subgroup analysis was conducted based on publication year, with 2009 serving as the dividing point. This choice aligns with an update made by the American Academy of Neurology Quality Standards Subcommittee in 2009, which emphasized the importance of multidisciplinary care in optimizing healthcare services and extending the survival of patients with ALS [36]. Subgroup analysis revealed a significant increase in OS for trials published after 2009 compared with those published before 2009. These findings suggest that advances in medical conditions and care practices positively affect the survival of patients with ALS. Consequently, when utilizing historical comparisons, studies conducted after 2009 should be considered to obtain more relevant and accurate results.

The ALSFRS-R scores in the placebo group compared with the baseline values gradually decreased, taking approximately 17.5 months to reach 50% of the maximum reduction. Considering the ALS trials included in the present study, the treatment duration ranged between 3 and 48 months, during which the reduction of the placebo group approached 10.8% to 76.9% of its maximum value, with the reduced ALSFRS-R scores differing substantially. Therefore, the heterogeneity in treatment duration between trials should be considered when estimating the sample size of clinical trials or comparing the efficacy of cross-sectional studies. A real-world study suggests that riluzole slows the decline of ALSFRS-R scores [37]. However, our study did not find a significant effect of the percentage of riluzole use on ALSFRS-R scores, which may be related to the extent of riluzole's impact on ALSFRS-R and the detection power afforded by the volume of data included in this research. Further data accumulation is needed to confirm these findings.

The models established in the present study accurately predicted the OS distribution in the placebo group, considering different durations or percentages of riluzole-treated patients. Additionally, the established models could precisely estimate the distribution of the ALSFRS-R score in the placebo group under distinct treatment durations, which can provide precise external control for single-arm ALS studies and a basis for the preliminary assessment of drug efficacy. We evaluated the efficacy of lithium carbonate and VM202 for the treatment of ALS based on the established disease course in the placebo group. Lithium carbonate, commonly used to treat neuropsychiatric illnesses by mediating its action via lithium ions, is primarily used to treat mania and bipolar disorder [20]. VM202 is a plasmid DNA that expresses two subtypes of human hepatocyte growth factors that strongly induce angiogenesis and cell migration. As a novel gene therapy, VM202 has potential efficacy in treating ischemic heart disease and painful diabetic peripheral neuropathy [38]. Theoretically, these two drugs could be used to treat ALS. Compared with the response observed in the historical placebo group comprising patients with the same proportion of riluzole treatment and disease duration, our findings indicated that lithium carbonate did not improve the OS of patients with ALS. Additionally, there was no significant difference in the efficacy of VM202 on the ALSFRS-R compared with the historical placebo group. Moreover, these two drugs failed to exhibit any notable therapeutic advantages in ALS.

In addition to providing an external control for single-arm trials, the historical response of the placebo group can provide reliable a priori information for the accurate estimation of the disease course of the placebo group in randomized controlled trials with small sample sizes [39], thereby reducing sampling errors and accurately assessing the efficacy of the test drugs. A phase II trial with a small sample size has shown that talampanel-treated patients with ALS have a 30% reduction in ALSFRS-R compared with placebo-treated patients. Thus, talampanel may afford improved efficacy in ALS and might be promoted in phase III clinical trials. However, the sample size of the placebo group was only 19 patients, and the observed superior efficacy of talampanel may be attributed to sampling errors [22]. The present study used the responses of the historical placebo group on the ALSFRS-R as a priori information. The posterior distribution of the responses in the placebo group was accurately estimated. We found that talampanel did not significantly improve ALSFRS-R scores compared with the placebo. Borrowing distribution information from a historical placebo group is equivalent to expanding the sample size of the placebo group, which is particularly important considering the scarcity of clinical trial resources for rare diseases. The FDA has used a similar approach to treat systemic lupus erythematosus (SLE) in children using belimumab. Given the small number of children with SLE included in the trial, belimumab cannot be conclusively established as a significantly superior treatment to the placebo. Considering the efficacy of belimumab in treating adult SLE as a priori information, the FDA found that the efficacy of belimumab in children with SLE is significantly better than that of a placebo, borrowing 55% of the information from the efficacy observed in adults. Considering the similarity in disease and drug responses between children and adults with SLE, the FDA approved belimumab for the treatment of SLE in children [39]. By establishing a time course and covariate model, an MBMA can provide more accurate a priori information regarding the disease course of the historical placebo group than traditional meta-analysis, which is of considerable value for saving limited clinical trial resources for rare diseases and accelerating the research and development of orphan drugs. However, historical comparisons should be limited to small-sample exploratory trials and should not be considered as conclusive evidence. The median sample size of the placebo groups in the phase I and II clinical trials for ALS included in this study was 55. This indicates that when the sample size of a placebo group is below 55, incorporating data from historical placebo groups may be appropriate. However, it should be noted that the final conclusions should be validated using large-scale clinical trials.

This study had some limitations. Firstly, some clinically important factors, such as the rate of decline in the ALSFRS-R, slow vital capacity, and forced vital capacity, were not examined because of data limitations. Secondly, the definition of survival differed across trials. The definition of survival in the included studies can be roughly divided into three categories: the first category defines the termination of survival as patient death, the second category defines the termination of survival as patient death or the need for respiratory support (including tracheotomy and non-invasive ventilation), and the third category includes those without relevant definitions in the literature [40–42]. In this study, we conducted an exploratory analysis of the data (Additional file 1: Figure S4) and found no significant differences in OS among the three distinct definitions of survival. Finally, only studies published in English were included; therefore, the risk of publication bias cannot be excluded. 

## Conclusion

In this study, a quantitative analysis of the disease course and its influencing factors in a placebo group in ALS clinical trials was conducted. The findings revealed a significant association between OS in the placebo group and both disease course and the proportion of patients receiving riluzole treatment. The disease course model developed from historical placebo group data in this study offers a reliable external control for single-arm trials and provides valuable a priori information for accurately estimating placebo responses in small randomized controlled trials.

### Supplementary Information

**Additional file 1: Table S1.** Search strategy.** Table S2.** List of the included studies.** Table S3.** Brief characteristics of the included studies.** Table S4.** Summary of the included studies.** Figure S1.** Risk of bias assessment chart.** Figure S2.** Correlation analysis between covariates and model parameters.** Figure S3.** The goodness-of-fit of the final model of OS and ALSFRS-R.** Figure S4.** Overall survive data view with three different death definition.

## Data Availability

The data set supporting the results of this article are included within the article and other supplements.

## Abbreviations

ALS: Amyotrophic lateral sclerosis
ALSFRS-R: Amyotrophic Lateral Sclerosis Functional Rating Scale
CWRES: Conditional weighted residual errors
CIs: Confidence intervals
FDA: Food and Drug Administration
GOF: Goodness of fit
IPRED: Individual predictions
MBMA: Model-based meta-analysis
MU: Variance of the lognormal distribution
OFV: Objective functional value
OBS: Observations
OS: Overall survival
PRED: Population predictions
RSE: Relative standard error
RSD: Relative standardized deviation
N: Sample size
SIGM: SIGM, the median of the lognormal distribution
SLE: Systemic lupus erythematosus
SVC: Slow vital capacity
ET_50_: Time to achieve 50% of the maximum efficacy
Emax: Maximum effect value
FVC: Forced vital capacity
VPC: Visual predictive check
θ: Model parameter
ε: Residual error
η: Inter-group variation
γ: Shape parameter

## References

[1] Brown RH, Al-Chalabi A (2017). Amyotrophic lateral sclerosis. N Engl J Med.

[2] Niedermeyer S, Murn M, Choi PJ (2019). Respiratory failure in amyotrophic lateral sclerosis. Chest.

[3] Goutman SA, Hardiman O, Al-Chalabi A, Chió A, Savelieff MG, Kiernan MC (2022). Recent advances in the diagnosis and prognosis of amyotrophic lateral sclerosis. Lancet Neurol.

[4] Xu L, Liu T, Liu L, Yao X, Chen L, Fan D (2020). Global variation in prevalence and incidence of amyotrophic lateral sclerosis: a systematic review and meta-analysis. J Neurol.

[5] Masrori P, Van Damme P (2020). Amyotrophic lateral sclerosis: a clinical review. Eur J Neurol.

[6] Miller RG, Mitchell JD, Moore DH. Riluzole for amyotrophic lateral sclerosis (ALS)/motor neuron disease (MND). The Cochrane database of systematic reviews, 2012(3), CD001447. 10.1002/14651858.CD001447.pub3 PMC7055506 22419278

[7] Bensimon G, Lacomblez L, Meininger V (1994). A controlled trial of riluzole in amyotrophic lateral sclerosis. ALS/Riluzole Study Group. New Engl J Med.

[8] Kiernan MC, Vucic S, Talbot K, McDermott CJ, Hardiman O, Shefner JM (2021). Improving clinical trial outcomes in amyotrophic lateral sclerosis. Nat Rev Neurol.

[9] Paganoni S, Hendrix S, Dickson SP, Knowlton N, Macklin EA, Berry JD (2021). Long-term survival of participants in the CENTAUR trial of sodium phenylbutyrate-taurursodiol in amyotrophic lateral sclerosis. Muscle Nerve.

[10] Mandema JW, Gibbs M, Boyd RA, Wada DR, Pfister M (2011). Model-based meta-analysis for comparative efficacy and safety: application in drug development and beyond. Clin Pharmacol Ther.

[11] Demin I, Hamrén B, Luttringer O, Pillai G, Jung T (2012). Longitudinal model-based meta-analysis in rheumatoid arthritis: an application toward model-based drug development. Clin Pharmacol Ther.

[12] Food and Drug Administration. Amyotrophic lateral sclerosis: developing drugs for treatment, draft guidance for industry. Available at: fda.gov/downloads/Drugs/GuidanceComplianceRegulatoryInformation/Guidances/UCM596718.pdf. Accessed June 6, 2018.

[13] Andrews JA, Bruijn LI, Shefner JM (2019). ALS drug development guidances and trial guidelines: Consensus and opportunities for alignment. Neurology.

[14] Balendra R, Jones A, Jivraj N, Knights C, Ellis CM, Burman R (2014). Estimating clinical stage of amyotrophic lateral sclerosis from the ALS Functional Rating Scale. Amyotroph Lateral Scler Frontotemporal Degener.

[15] Gordon PH, Miller RG, Moore DH (2004). ALSFRS-R. Amyotroph Lateral Scler Motor Neuron Disord.

[16] Cumpston M, Li T, Page MJ, Chandler J, Welch VA, Higgins JP et al. Updated guidance for trusted systematic reviews: a new edition of the Cochrane Handbook for Systematic Reviews of Interventions. Cochrane Database Syst Rev. 2019;10:Ed000142. 10.1002/14651858.ED000142 PMC10284251 31643080

[17] Hooker AC, Staatz CE, Karlsson MO (2007). Conditional weighted residuals (CWRES): a model diagnostic for the FOCE method. Pharm Res.

[18] Wang DD, Zhang S (2012). Standardized visual predictive check versus visual predictive check for model evaluation. J Clin Pharmacol.

[19] Thai HT, Mentré F, Holford NH, Veyrat-Follet C, Comets E (2014). Evaluation of bootstrap methods for estimating uncertainty of parameters in nonlinear mixed-effects models: a simulation study in population pharmacokinetics. J Pharmacokinet Pharmacodyn.

[20] Chiò A, Borghero G, Calvo A, Capasso M, Caponnetto C, Corbo M (2010). Lithium carbonate in amyotrophic lateral sclerosis: lack of efficacy in a dose-finding trial. Neurology.

[21] Sufit RL, Ajroud-Driss S, Casey P, Kessler JA (2017). Open label study to assess the safety of VM202 in subjects with amyotrophic lateral sclerosis. Amyotrop Lateral Scler Frontotemporal Degener.

[22] Pascuzzi RM, Shefner J, Chappell AS, Bjerke JS, Tamura R, Chaudhry V (2010). A phase II trial of Talampanel in subjects with amyotrophic lateral sclerosis. Amyotroph Lateral Scler.

[23] Bovis F, Ponzano M, Signori A, Schiavetti I, Bruzzi P, Sormani MP (2022). Reinterpreting clinical trials in children with multiple sclerosis using a Bayesian approach. JAMA Neurol.

[24] Miller RG, Mitchell JD, Lyon M, Moore DH (2003). Riluzole for amyotrophic lateral sclerosis (ALS)/motor neuron disease (MND). Amyotrop Lateral Scler Motor Neuron Disord.

[25] Tavazzi E, Gatta R, Vallati M, Cotti Piccinelli S, Filosto M, Padovani A, et al. Leveraging process mining for modeling progression trajectories in amyotrophic lateral sclerosis. BMC Med Inform Decis Mak. 2023;22(Suppl 6):346. Published 2023 Feb 2. 7 10.1186/s12911-023-02113-7 PMC9896660 36732801

[26] van Eenennaam RM, Kruithof WJ, van Es MA, Kruitwagen-van Reenen ET, Westeneng HJ, Visser-Meily JMA (2020). Discussing personalized prognosis in amyotrophic lateral sclerosis: development of a communication guide. BMC Neurol.

[27] De Marchi F, Mareschi K, Ferrero I, Cantello R, Fagioli F, Mazzini L (2023). Effect of mesenchymal stromal cell transplantation on long-term survival in amyotrophic lateral sclerosis. Cytotherapy.

[28] Gold J, Rowe DB, Kiernan MC, Vucic S, Mathers S, van Eijk RPA (2019). Safety and tolerability of Triumeq in amyotrophic lateral sclerosis: the Lighthouse trial. Amyotroph Lateral Scler Frontotemporal Degener.

[29] Paganoni S, Quintana M, Sherman AV, Vestrucci M, Wu Y, Timmons J, et al. Pooled Resource Open-Access ALS Clinical Trials Consortium. Analysis of sodium phenylbutyrate and taurursodiol survival effect in ALS using external controls. Ann Clin Transl Neurol. 2023;10(12):2297–2304. 10.1002/acn3.51915 PMC10723227 37807839

[30] Nam JY, Chun S, Lee TY, Seo Y, Kim K, Park J (2023). Long-term survival benefits of intrathecal autologous bone marrow-derived mesenchymal stem cells (Neuronata-R®: lenzumestrocel) treatment in ALS: propensity-score-matched control, surveillance study. Front Aging Neurosci.

[31] Song Y, Cheng H, Liu J, Kazuo S, Feng L, Wei Y, et al. Pooled Resource Open-Access ALS Clinical Trials Consortium. Effectiveness of herbal medicine on patients with amyotrophic lateral sclerosis: analysis of the PRO-ACT data using propensity score matching. Phytomedicine. 2022;107:154461.

[32] Su WM, Cheng YF, Jiang Z, Duan QQ, Yang TM, Shang HF (2021). Predictors of survival in patients with amyotrophic lateral sclerosis: a large meta-analysis. EBioMedicine.

[33] Kollewe K, Mauss U, Krampfl K, Petri S, Dengler R, Mohammadi B (2008). ALSFRS-R score and its ratio: a useful predictor for ALS-progression. J Neurol Sci.

[34] Su WM, Cheng YF, Jiang Z, Duan QQ, Yang TM, Shang HF, Chen YP (2021). Predictors of survival in patients with amyotrophic lateral sclerosis: a large meta-analysis. EBioMedicine.

[35] Chiò A, Logroscino G, Hardiman O, Swingler R, Mitchell D, Beghi E (2009). Prognostic factors in ALS: a critical review. Amyotrop Lateral Scler.

[36] Miller RG, Jackson CE, Kasarskis EJ, England JD, Forshew D, Johnston W (2009). Practice parameter update: the care of the patient with amyotrophic lateral sclerosis: multidisciplinary care, symptom management, and cognitive/behavioral impairment (an evidence-based review): report of the Quality Standards Subcommittee of the American Academy of Neurology. Neurology.

[37] Chen L, Liu X, Tang L, Zhang N, Fan D (2016). Long-term use of Riluzole could improve the prognosis of sporadic amyotrophic lateral sclerosis patients: a real-world cohort study in China. Front Aging Neurosci.

[38] Kessler JA, Shaibani A, Sang CN, Christiansen M, Kudrow D, Vinik A (2021). Gene therapy for diabetic peripheral neuropathy: a randomized, placebo-controlled phase III study of VM202, a plasmid DNA encoding human hepatocyte growth factor. Clin Transl Sci.

[39] Brunner HI, Abud-Mendoza C, Viola DO, Calvo Penades I, Levy D, Anton J (2020). Safety and efficacy of intravenous belimumab in children with systemic lupus erythematosus: results from a randomised, placebo-controlled trial. Ann Rheum Dis.

[40] Mora JS, Bradley WG, Chaverri D, Hernández-Barral M, Mascias J, Gamez J (2021). Long-term survival analysis of masitinib in amyotrophic lateral sclerosis. Ther Adv Neurol Disord.

[41] Statland JM, Moore D, Wang Y, Walsh M, Mozaffar T, Elman L (2019). Rasagiline for amyotrophic lateral sclerosis: a randomized, controlled trial. Muscle Nerve.

[42] Ludolph AC, Schuster J, Dorst J, Dupuis L, Dreyhaupt J, Weishaupt JH (2018). Safety and efficacy of rasagiline as an add-on therapy to riluzole in patients with amyotrophic lateral sclerosis: a randomised, double-blind, parallel-group, placebo-controlled, phase 2 trial. Lancet Neurol.

