# Supplementary material for: Accelerating drug development for amyotrophic lateral sclerosis: construction and application of a disease course model using historical placebo group data
Source: Orphanet J Rare Dis. 2024 Feb 2;19:40. doi: 10.1186/s13023-024-03057-5 (PMC10837960; doi:10.1186/s13023-024-03057-5)
Supplement: Supplementary file 1 — Additional file 1: Table S1. Search strategy. Table S2. List of the included studies. Table S3. Brief characteristics of the included studies. Table S4. Summary of the included studies. Figure S1. Risk of bias assessment chart. Figure S2. Correlation analysis between covariates and model parameters. Figure S3. The goodness-of-fit of the final model of OS and ALSFRS-R. Figure S4. Overall survive data view with three different death definition. [file 13023_2024_3057_MOESM1_ESM.docx]

**Supplementary Material**

**Method 1 Literature search strategy**

**Method 2 Model building**

**Table S1 Search strategy**

**Table S2 List of the included studies**

**Table S3 Summary of the included studies**

**Table S4 Covariate screening for inclusion**

**Figure S1 Risk of bias assessment chart**

**Figure S2 Correlation analysis between covariates and model parameters**

**Figure S3 The goodness-of-fit of the final model of OS and ALSFRS-R**

**Figure S4 Overall survival data view with three different death definition**

**Method 1 Literature search strategy**

A comprehensive search of PubMed and Cochrane Library databases was performed until February 21, 2022. The keywords were "Amyotrophic lateral sclerosis", The full search strategy is appended below, citations were downloaded in Endnote X9, and duplicates were screened and manually excluded by Endnote X9. Two researchers were involved in the inclusion of literature, reading the full text of potential studies and creating tables for data extraction.

**Tabel S1 Search strategy**

**Pubmed**

| No. | Query | Results | Date |
| --- | --- | --- | --- |
| #1 | Amyotrophic lateral sclerosis[Title/Abstract] | 24004 | 2022.2.21 |
| #2 | Filters: Clinical Trial | 563 | 2022.2.21 |

**Cochrane library**

| No. | Query | Results | Date |
| --- | --- | --- | --- |
| #1 | Amyotrophic lateral sclerosis [Title, Abstract, keywords] | 1352 | 2022.2.21 |
| #2 | Filters: Limited to trials | 1352 | 2022.2.21 |

**Method 2 Model building.**

**Structural model establishment**

In this study, two indicators, OS and the mean change from baseline in ALSFRS-R score, will be modeled and analyzed. Parametric survival models were used to analyze OS data. The survival model is related to the hazard function h(t), which can be interpreted as the instantaneous risk of death at moment t. The survival function and the hazard function are related as follows:

$S(t)=exp(-\int_{0}^{t} h(t)dt)$ (1)

In Equation 1, S(t) is the survival rate at time point t, $\int_{0}^{t} h(t)dt$ is the cumulative risk of death from time point 0 to time point t.

To select an appropriate structure model, four different hazard functions were evaluated (Equation 2-5). In Equation 2-4, the parameters λ and β represent the hazard rate at time 0 and the regression coefficient of the hazard rate changing over time, respectively. While in Eq. 5, µ and σ are the medians and standard deviation of the lognormal distribution, which control the shape of the function curve.

Constant: h(t)= λ (2)

Gompert: h(t)= λ**·**exp(β**·**t) (3)

Weibull: h(t)= λ**·**exp(β**·**ln(t)) (4)

Lognormal: $h\left( t \right)=\frac{{(\sigma t\sqrt{2\pi})}^{-1}e^{{(-\frac{1}{2}z}^{2})}}{1-\emptyset(z)}, Z=\frac{\ln\left( t \right)-\mu}{\sigma}$ (5)

The selection of the four hazard functions was based on the minimum value of the objective function value (OFV) provided by the NONMEM software, the relative standard errors of the model parameter estimates, and the goodness of fit plots.

The mean change from baseline in ALSFRS-R score gradually increased over time and eventually plateaued. The distributional characteristics of these data could be described by the Sigmoid Emax model (Equation 6).

**$\text{E}_{t}=\frac{\text{E}_{\text{max}}\times\text{time}^{\text{γ}}}{\text{ET}_{\text{50 }}^{\text{γ}}+\text{time}^{\text{γ}}}$** (6)

In Equation 6, E_max_ represented the maximum effect, ET_50_ was the onset time, and γ is the shape parameter.

**Random effects model establishment**

Inter-trial variation and residual error were added to the structure model to account for the variation between the observed value and the model-predicted typical values. Inter-trial variation is introduced into the model parameters in exponential form (Equation 7), and residual error is described by the additive model (Equation 8).

$\text{P}_{\text{i}}=\text{P}_{\text{pop}}\times\exp(\text{η}_{i})$ (7)

${Obs}_{i,j}={Pred}_{i,j}+w_{i,j}\times\varepsilon_{i,j}$ (8)

 (9)

In Equations 7, P_i_ is the individual prediction of the model parameter, P_pop_ is the population prediction of the corresponding model parameter. η_i_ represents the interstudy variability, which is assumed to be normally distributed, with a mean of 0 and variance of ω_i_^2^.

In Equation 8, Obs_i,j_ and Pred_i,j_ represent the observed and predicted value at time point j of study i, respectively. ɛ_i,j_ is a residual at time point j of study i, which is assumed to be normally distributed with a mean of 0 and variance of σ^2^. The residual error was weighed by the inverse square root of the corresponding sample size N_i,j_ at that time point (Equation 9).

**Covariate model establishment**

The potential factors influencing the model parameters were investigated, including the age of onset, proportion of males, disease duration, baseline of ALSFRS-R, the year of publication, the proportion of patients treated with riluzole, and the proportion of patients receiving basic treatment, proportion of individuals with bulbar onset. For factors with a missing proportion of less than 30%, the median was used to fill in the missing values. Factors with ≥30% missing data were not considered, and missing values for factors with less than 30% missing data were imputed with the median.

For the OS model, the covariates were introduced into the hazard function according to the following formula:

$h(t){\_}_{Final}=h(t){\_}_{Base}\cdot e^{(\alpha_{1}\cdot x_{1}+\alpha_{2}\cdot x_{2}+\cdots+\alpha_{n}\cdot x_{n})}$ (10)

In Equation 10, $e^{(\alpha_{1}\cdot x_{1}+\alpha_{2}\cdot x_{2}+\cdots+\alpha_{n}\cdot x_{n})}$describes the influence of covariates on the risk of death. Here, x_1_-x_n_, represent the values of n covariates, and their coefficients of influence are are α_1_-α_n_.

For the model of ALSFRS-R, the categorical variables were introduced using Equation 11, and continuous variables were introduced using Equation 12.

$P_{pop}=P_{Typical}+COV\times\theta_{cov}$ (11)

$P_{pop}=P_{Typical}\times(COV/COV_{median})^{\theta cov}$ (12)

In Equations 11–12, P_pop_ is the population value of model parameter corresponding to the different covariate levels, P_Typical_ is the typical value of model parameter when the categorical covariate is equal to 0 or continuous covariate is equal to COV_median_. COV is the covariate value, COV_median_ is the median of the covariate, and θ is the coefficient of the covariate for the model parameter.

The covariates were tested using the forward inclusion and backward elimination method and also consider the clinical implications. Upon introduction of a covariate, a decrease in OFV of 3.84 (χ^2^, α = 0.05, df = 1) was considered statistically significant in the forward inclusion process. During the backward elimination procedure, a covariate was removed from the model if the OFV increased < 6.63 (χ^2^, α = 0.01, df= 1) during the exclusion.

**The expression of the final model**

The covariate model of OS in placebo group can be expressed in Formula (13)

$h\left( t \right)=h(t){\_}_{\mathrm{Base}}*e^{\left( DURATION-17.8 \right)*0.0317-RILUZOLE*0.228}$ (13)

In Equation 1, h(t)__Base_ is the base hazard function, DURATION indicates the time from first symptom onset to enrollment, 17.8 is the median duration of the patients, RILUZOLE indicates the percentage of patients receiving riluzole treatment, and 0.0317 and -0.228 are covariate coefficients for the hazard function.

**Table S2 List of the included studies**

1. Mora JS, Bradley WG, Chaverri D, Hernandez-Barral M, Mascias J, Gamez J, et al. Long-term survival analysis of masitinib in amyotrophic lateral sclerosis. Therapeutic advances in neurological disorders. 2021;14.

2. Statland JM, Moore D, Wang Y, Walsh M, Mozaffar T, Elman L, et al. Rasagiline for amyotrophic lateral sclerosis: a randomized, controlled trial. Muscle & nerve. 2019;59(2):201‐7.

Amyotrophic lateral sclerosis & frontotemporal degeneration. 2019;20(1-2):115-22.

3. Ludolph AC, Schuster J, Dorst J, Dupuis L, Dreyhaupt J, Weishaupt JH, et al. Safety and efficacy of rasagiline as an add-on therapy to riluzole in patients with amyotrophic lateral sclerosis: a randomised, double-blind, parallel-group, placebo-controlled, phase 2 trial. The Lancet Neurology. 2018;17(8):681‐8.

4. Ahmadi M, Agah E, Nafissi S, Jaafari MR, Harirchian MH, Sarraf P, et al. Safety and Efficacy of Nanocurcumin as Add-On Therapy to Riluzole in Patients With Amyotrophic Lateral Sclerosis: a Pilot Randomized Clinical Trial. Neurotherapeutics : the journal of the American Society for Experimental NeuroTherapeutics. 2018;15(2):430‐8.

5. Meininger V, Genge A, van den Berg LH, Robberecht W, Ludolph A, Chio A, et al. Safety and efficacy of ozanezumab in patients with amyotrophic lateral sclerosis: a randomised, double-blind, placebo-controlled, phase 2 trial. The Lancet Neurology. 2017;16(3):208-16.

6. Elia AE, Lalli S, Monsurrò MR, Sagnelli A, Taiello AC, Reggiori B, et al. Tauroursodeoxycholic acid in the treatment of patients with amyotrophic lateral sclerosis. European journal of neurology. 2016;23(1):45‐52.

7. Lauria G, Dalla Bella E, Antonini G, Borghero G, Capasso M, Caponnetto C, et al. Erythropoietin in amyotrophic lateral sclerosis: a multicentre, randomised, double blind, placebo controlled, phase III study. Journal of neurology, neurosurgery, and psychiatry. 2015;86(8):879‐86.

8. Dupuis L, Dengler R, Heneka MT, Meyer T, Zierz S, Kassubek J, et al. A randomized, double blind, placebo-controlled trial of pioglitazone in combination with riluzole in amyotrophic lateral sclerosis. PloS one. 2012;7(6):e37885.

9. Lenglet T, Lacomblez L, Abitbol JL, Ludolph A, Mora JS, Robberecht W, et al. A phase II-III trial of olesoxime in subjects with amyotrophic lateral sclerosis. European journal of neurology. 2014;21(3):529‐36.

10. Cudkowicz ME, Titus S, Kearney M, Yu H, Sherman A, Schoenfeld D, et al. Safety and efficacy of ceftriaxone for amyotrophic lateral sclerosis: a multi-stage, randomised, double-blind, placebo-controlled trial. The Lancet Neurology. 2014;13(11):1083-91.

11. Verstraete E, Veldink JH, Huisman MH, Draak T, Uijtendaal EV, van der Kooi AJ, et al. Lithium lacks effect on survival in amyotrophic lateral sclerosis: a phase IIb randomised sequential trial. Journal of neurology, neurosurgery, and psychiatry. 2012;83(5):557‐64.

12. Piepers S, Veldink JH, de Jong SW, van der Tweel I, van der Pol WL, Uijtendaal EV, et al. Randomized sequential trial of valproic acid in amyotrophic lateral sclerosis. Annals of neurology. 2009;66(2):227‐34.

13. Meininger V, Drory VE, Leigh PN, Ludolph A, Robberecht W, Silani V. Glatiramer acetate has no impact on disease progression in ALS at 40 mg/day: a double- blind, randomized, multicentre, placebo-controlled trial. Amyotrophic lateral sclerosis. 2009;10(5‐6):378‐83.

14. Lauria G, Campanella A, Filippini G, Martini A, Penza P, Maggi L, et al. Erythropoietin in amyotrophic lateral sclerosis: a pilot, randomized, double-blind, placebo-controlled study of safety and tolerability. Amyotrophic lateral sclerosis. 2009;10(5‐6):410‐5.

15. Sorenson EJ, Windbank AJ, Mandrekar JN, Bamlet WR, Appel SH, Armon C, et al. Subcutaneous IGF-1 is not beneficial in 2-year ALS trial. Neurology. 2008;71(22):1770‐5.

16. Rosenfeld J, King RM, Jackson CE, Bedlack RS, Barohn RJ, Dick A, et al. Creatine monohydrate in ALS: effects on strength, fatigue, respiratory status and ALSFRS. Amyotrophic lateral sclerosis. 2008;9(5):266‐72.

17. Gordon PH, Moore DH, Miller RG, Florence JM, Verheijde JL, Doorish C, et al. Efficacy of minocycline in patients with amyotrophic lateral sclerosis: a phase III randomised trial. The Lancet Neurology. 2007;6(12):1045‐53.

18. Meininger V, Asselain B, Guillet P, Leigh PN, Ludolph A, Lacomblez L, et al. Pentoxifylline in ALS: a double-blind, randomized, multicenter, placebo-controlled trial. Neurology. 2006;66(1):88‐92.

19. Groeneveld GJ, Veldink JH, van der Tweel I, Kalmijn S, Beijer C, de Visser M, et al. A randomized sequential trial of creatine in amyotrophic lateral sclerosis. Annals of neurology. 2003;53(4):437‐45.

20. Miller RG, Moore DH, Gelinas DF, Dronsky V, Mendoza M, Barohn RJ, et al. Phase III randomized trial of gabapentin in patients with amyotrophic lateral sclerosis. Neurology. 2001;56(7):843‐8.

21. Desnuelle C, Dib M, Garrel C, Favier A. A double-blind, placebo-controlled randomized clinical trial of alpha-tocopherol (vitamin E) in the treatment of amyotrophic lateral sclerosis. ALS riluzole-tocopherol Study Group. Amyotrophic lateral sclerosis and other motor neuron disorders. 2001;2(1):9‐18.

22. Bradley WG. A controlled trial of recombinant methionyl human BDNF in ALS. Neurology. 1999;52(7):1427‐33.

23. Gredal O, Werdelin L, Bak S, Christensen PB, Boysen G, Kristensen MO, et al. A clinical trial of dextromethorphan in amyotrophic lateral sclerosis. Acta neurologica Scandinavica. 1997;96(1):8‐13.

24. Bensimon G, Lacomblez L, Meininger V. A controlled trial of riluzole in amyotrophic lateral sclerosis. ALS/Riluzole Study Group. New England journal of medicine. 1994;330(9):585‐91.

25. Lacomblez, L., Bensimon, G., Leigh, P. N., Guillet, P., Powe, L., Durrleman, S., et al. A confirmatory dose-ranging study of riluzole in ALS. ALS/Riluzole Study Group-II. Neurology, 1996;47(6), 242–250.

26. Louwerse ES, Weverling GJ, Bossuyt PM, Meyjes FE, de Jong JM. Randomized, double-blind, controlled trial of acetylcysteine in amyotrophic lateral sclerosis. Archives of neurology. 1995;52(6):559‐64.

27. Eisen A, Stewart H, Schulzer M, Cameron D. Anti-glutamate therapy in amyotrophic lateral sclerosis: a trial using lamotrigine. Canadian journal of neurological sciences [Journal canadien des sciences neurologiques]. 1993;20(4):297‐301.

28. Branched-chain amino acids and amyotrophic lateral sclerosis: a treatment failure? The Italian ALS Study Group. Neurology. 1993;43(12):2466‐70.

29. Cudkowicz ME, van den Berg LH, Shefner JM, Mitsumoto H, Mora JS, Ludolph A, et al. Dexpramipexole versus placebo for patients with amyotrophic lateral sclerosis (EMPOWER): a randomised, double-blind, phase 3 trial. The Lancet Neurology. 2013;12(11):1059-67.

30. Beghi E, Pupillo E, Bonito V, Buzzi P, Caponnetto C, Chiò A, et al. Randomized double-blind placebo-controlled trial of acetyl-L-carnitine for ALS. Amyotrophic lateral sclerosis & frontotemporal degeneration. 2013;14(5‐6):397‐405.

31. Dalla Bella E, Bersano E, Antonini G, Borghero G, Capasso M, Caponnetto C, et al. The unfolded protein response in amyotrophic later sclerosis: results of a phase 2 trial. Brain : a journal of neurology. 2021;144(9):2635-47.

32. Kaji R, Imai T, Iwasaki Y, Okamoto K, Nakagawa M, Ohashi Y, et al. Ultra-high-dose methylcobalamin in amyotrophic lateral sclerosis: a long-term phase II/III randomised controlled study. Journal of neurology, neurosurgery, and psychiatry. 2019;90(4):451‐7.

33. de la Rubia JE, Drehmer E, Platero JL, Benlloch M, Caplliure-Llopis J, Villaron-Casales C, et al. Efficacy and tolerability of EH301 for amyotrophic lateral sclerosis: a randomized, double-blind, placebo-controlled human pilot study.

34. Safety and efficacy of edaravone in well defined patients with amyotrophic lateral sclerosis: a randomised, double-blind, placebo-controlled trial. The Lancet Neurology. 2017;16(7):505‐12.

35. Amirzagar, N., Nafissi, S., Tafakhori, A., Modabbernia, A., Amirzargar, A., Ghaffarpour, M., Siroos, B., & Harirchian, M. H. (2015). Granulocyte colony-stimulating factor for amyotrophic lateral sclerosis: a randomized, double-blind, placebo-controlled study of Iranian patients. Journal of clinical neurology (Seoul, Korea),

36. Abe K, Itoyama Y, Sobue G, Tsuji S, Aoki M, Doyu M, et al. Confirmatory double-blind, parallel-group, placebo-controlled study of efficacy and safety of edaravone (MCI-186) in amyotrophic lateral sclerosis patients. Amyotrophic lateral sclerosis & frontotemporal degeneration. 2014;15(7‐8):610‐7.

37. Morrison KE, Dhariwal S, Hornabrook R, Savage L, Burn DJ, Khoo TK, et al. Lithium in patients with amyotrophic lateral sclerosis (LiCALS): a phase 3 multicentre, randomised, double-blind, placebo-controlled trial. The Lancet Neurology. 2013;12(4):339‐45.

38. Cudkowicz M, Bozik ME, Ingersoll EW, Miller R, Mitsumoto H, Shefner J, et al. The effects of dexpramipexole (KNS-760704) in individuals with amyotrophic lateral sclerosis. Nature medicine. 2011;17(12):1652-6.

39. Pascuzzi RM, Shefner J, Chappell AS, Bjerke JS, Tamura R, Chaudhry V, et al. A phase II trial of talampanel in subjects with amyotrophic lateral sclerosis. Amyotrophic lateral sclerosis. 2010;11(3):266-71.

40. Nefussy B, Artamonov I, Deutsch V, Naparstek E, Nagler A, Drory VE. Recombinant human granulocyte-colony stimulating factor administration for treating amyotrophic lateral sclerosis: a pilot study. Amyotrophic lateral sclerosis. 2010;11(1‐2):187‐93.

41. Aggarwal SP, Zinman L, Simpson E, McKinley J, Jackson KE, Pinto H, et al. Safety and efficacy of lithium in combination with riluzole for treatment of amyotrophic lateral sclerosis: a randomised, double-blind, placebo-controlled trial. The Lancet Neurology. 2010;9(5):481-8.

42. Cudkowicz ME, Shefner JM, Schoenfeld DA, Zhang H, Andreasson KI, Rothstein JD, et al. Trial of celecoxib in amyotrophic lateral sclerosis. Annals of neurology. 2006;60(1):22‐31.

43. Shefner JM, Cudkowicz ME, Schoenfeld D, Conrad T, Taft J, Chilton M, et al. A clinical trial of creatine in ALS. Neurology. 2004;63(9):1656‐61.

44. Weiss MD, Macklin EA, Simmons Z, Knox AS, Greenblatt DJ, Atassi N, et al. A randomized trial of mexiletine in ALS: Safety and effects on muscle cramps and progression. Neurology. 2016;86(16):1474-81.

45. Saccà F, Quarantelli M, Rinaldi C, Tucci T, Piro R, Perrotta G, et al. A randomized controlled clinical trial of growth hormone in amyotrophic lateral sclerosis: clinical, neuroimaging, and hormonal results. Journal of neurology. 2012;259(1):132‐8.

46. Kaufmann P, Thompson JL, Levy G, Buchsbaum R, Shefner J, Krivickas LS, et al. Phase II trial of CoQ10 for ALS finds insufficient evidence to justify phase III. Annals of neurology. 2009;66(2):235‐44.

47. Vucic S, Henderson RD, Mathers S, Needham M, Schultz D, Kiernan MC. Safety and efficacy of dimethyl fumarate in ALS: randomised controlled study. Annals of clinical and translational neurology. 2021.

**Table S3 Summary of the included studies**

| **Overall survival** | Intervention | Dose | Location | Duration,  month | Sample  size | Age,  year | Use of  rizuloze% | Baseline of  ALSFRS-R | Definition of Survival |
| --- | --- | --- | --- | --- | --- | --- | --- | --- | --- |
| Mora 2021^[1]^ | Masitinib | 4.5mg/kg | International | 12 | 130 | 55.5±10.6 | NA | 37.5 | NA |
|  | Placebo | - |  |  | 133 | 55.2±10.6 | NA | 38.1 |  |
|  |  |  |  |  |  |  |  |  |  |
| Statland 2019^[2]^ | Rasagiline | 2mg | USA | 12 | 60 | 58.4±10.2 | 82 | 38.2 | Death or use of invasive ventilation |
|  | Placebo | - |  |  | 20 | 57.5±8.5 | 80 | 35.9 |  |
|  |  |  |  |  |  |  |  |  |  |
| Ludolph 2018^[3]^ | Rasagiline | 100mg | German | 18 | 126 | 60.1±11.2 | 100 | 37.9 | Death or the study  cut-off date |
|  | Placebo | - |  |  | 125 | 60.4±10.2 | 100 | 38.3 |  |
|  |  |  |  |  |  |  |  |  |  |
| Ahmadi 2018^[4]*^ | Nanocurcumin | 80mg | Iran | 12 | 27 | 51.5±13.1 | 100 | 36 | Death or mechanical ventilation dependency |
|  | Placebo | - |  |  | 27 | 58.5±9.6 | 100 | 32.6 |  |
|  |  |  |  |  |  |  |  |  |  |
| Meininger 2017^[5]*^ | Ozanezumab | 15 mg/kg | Europe | 24 | 152 | 55.7±10.4 | 86 | 37.7 | Death or censoring |
|  | Placebo | - |  |  | 151 | 55.5±11.0 | 87 | 38.4 |  |
|  |  |  |  |  |  |  |  |  |  |
| Elia 2016^[6]*^ | TUDCA | 1000mg | Italy | 13.5 | 15 | 54.0±12.2 | 100 | 38.7 | NA |
|  | Placebo | - |  |  | 14 | 58.2±12.9 | 100 | 38.4 |  |
|  |  |  |  |  |  |  |  |  |  |
| Lauria 2015^[7]*^ | Erythropoietin | 371.2mg | Italy | 12 | 103 | 59.4±9.7 | 97.1 | 38.4 | Death,tracheotomy or >23 h NIV daily for 14 days |
|  | Placebo | - |  |  | 97 | 58.6±10.5 | 94.9 | 38.3 |  |
|  |  |  |  |  |  |  |  |  |  |
|  |  |  |  |  |  |  |  |  |  |
| Dupuis 2012^[8]*^ | Pioglitazone | 45mg | German | 18 | 109 | 58.9±10.6 | 100 | 37.5 | Death or last patient contact |
|  | Placebo |  |  |  | 109 | 59.0±10.4 | 100 | 37.0 |  |
|  |  | - |  |  |  |  |  |  |  |
| Lenglet 2014^[9]*^ | Olesoxime | 660mg | France | 18 | 259 | 57.3±11.2 | 100 | 39.1 | Permanent NIV ( >23 h daily for 14 consecutive days) or tracheostomy |
|  | Placebo | - |  |  | 253 | 55.7±11.2 | 100 | 38.2 |  |
|  |  |  |  |  |  |  |  |  |  |
|  |  |  |  |  |  |  |  |  |  |
| Cudkowicz 2014^[10]^ | Ceftriaxone | 2~4000mg | USA | 12 | 340 | 56±10 | 73 | 36.5 | Death, tracheostomy, or initiation of permanent assisted ventilation |
|  | Placebo | - |  |  | 173 | 55±10 | 74 | 36.9 |  |
|  |  |  |  |  |  |  |  |  |  |
|  |  |  |  |  |  |  |  |  |  |
| Verstraete 2012^[11]*^ | Lithium | 5mg | Netherlands | 16 | 66 | NA | 100 | NA | Death, tracheostomal ventilation or NIV for more than 16 h/day |
|  | Placebo | - |  |  | 67 | NA | 100 | NA |  |
|  |  |  |  |  |  |  |  |  |  |
|  |  |  |  |  |  |  |  |  |  |
| Piepers 2009^[12]*^ | Valproic acid | 1500mg | NL | 12 | 82 | NA | 100 | NA | Death or tracheostomy or NIV more than 23 hours/day |
|  | Placebo | - | - |  | 81 | NA | 100 | NA |  |
|  |  |  |  |  |  |  |  |  |  |
|  |  |  |  |  |  |  |  |  |  |
| Meininger 2009^[13]*^ | Glatiramer acetate | 40mg | USA |  | 184 | 55.7±11.7 | 100 | 38.7 | Death, tracheostomy or  PAV |
|  | Placebo |  |  |  | 182 | 56.7±12.1 | 100 | 38.1 |  |
|  |  |  |  |  |  |  |  |  |  |
| Lauria 2009^[14]*^ | Erythropoietin | 2857.1IU | UK | 24 | 12 | 53±8.6 | 100 | NA | Death or tracheotomy |
|  | Placebo |  |  |  | 11 | 58±10.6 | 100 | NA |  |
|  |  |  |  |  |  |  |  |  |  |
| Sorenson 2008^[15]^ | IGF-1 | 0.05mg/kg | NA | 24 | 167 | 53.9 | 69.5 | 38.7 | Death or tracheotomy |
|  | Placebo |  |  |  | 163 | 54.8 | 69.3 | 38.7 |  |
|  |  |  |  |  |  |  |  |  |  |
| Rosenfeld 2008^[16]^ | Creatine | 5000mg | UK | 9 | 53 | 56±10 | NA | 36 | NA |
|  | Placebo | - |  |  | 54 | 59±11 | NA | 36 |  |
|  |  |  |  |  |  |  |  |  |  |
| Gordon 2007^[17]^ | Minocycline | 400mg | USA | 9 | 206 | 58.6±11.8 | 67 | 37.8 | Death, tracheostomy, chronic assisted ventilation, or >23 h of NIV daily for 14 days |
|  | Placebo | - |  |  | 206 | 57.7±10.9 | 66 | 37.9 |  |
|  |  | - |  |  |  |  |  |  |  |
|  |  |  |  |  |  |  |  |  |  |
| Meininger 2006^[18]^ | Pentoxifylline | 1200mg | Europe | 18 | 199 | 57.1±11.7 | 100 | 38.9 | Death or ventilatory support |
|  | Placebo | - |  |  | 201 | 56.7±12.1 | 100 | 38.9 |  |
|  |  |  |  |  |  |  |  |  |  |
| Groeneveld 2003^[19]^ | Creatine | 10mg | Netherlands | 12 | 88 | 57.1±11.4 | 100 | 30.25 | Death, persistent assisted ventilation, or tracheostomy |
|  | Placebo | - |  |  | 87 | 58.4±10.7 | 100 | 30.74 |  |
|  |  |  |  |  |  |  |  |  |  |
|  |  |  |  |  |  |  |  |  |  |
| Miller 2001^[20]^ | Gabapentin | 3600mg | USA | 9 | 102 | 61.3±13.1 | 0 | 28.6 | NA |
|  | Placebo |  |  |  | 102 | 62±12.1 | 0 | 41.65 |  |
|  |  |  |  |  |  |  |  |  |  |
| Desnuelle 2001^[21]^ | Vitamin E | 1000mg | France | 12 | 144 | 62.5±11.2 | 100 | NA | Death or tracheostomy |
|  | Placebo |  |  |  | 144 | 65.7±9.4 | 100 | NA |  |
|  |  | - |  |  |  |  |  |  |  |
| Bradley 1999^[22]^ | BDNF | 25/100 µg/kg | North America | 9 | 748 | 55.95±12.7 | 2.7 | 30.0 | NA |
|  | Placebo | - |  |  | 387 | 55.9±12.4 | 3.9 | 30.1 |  |
|  |  |  |  |  |  |  |  |  |  |
| Gredal 1997^[23]^ | Dextromethorphan | 150mg | Denmark | 12 | 22 | 60±9.5 | 0 | NA | Death |
|  | Placebo |  |  |  | 23 | 58±9.8 | 0 | NA |  |
|  |  |  |  |  |  |  |  |  |  |
| Bensimon1994^[24]^ | Riluzole | 100mg | USA | 12 | 77 | 56.8±11 | 100 | NA | Death or tracheostomy |
|  | Placebo |  |  |  | 78 | 58.1±11 | 0 | NA |  |
|  |  |  |  |  |  |  |  |  |  |
| Lacomblez 1996^[25]^ | Riluzole | 50/100/200 mg | France | 18 | 717 | NA | 100 | NA | Death, tracheostomy, or intubation with artificial ventilation |
|  | Placebo |  |  |  | 242 | NA | 0 | NA |  |
|  |  |  |  |  |  |  |  |  |  |
|  |  |  |  |  |  |  |  |  |  |
| Louwerse 1995^[26]^ | Acetylcysteine | 50mg/kg | USA | 12 | 54 | 58±11 | 0 | NA | Death, long-term assisted ventilation, tracheostomy |
|  | Placebo |  |  |  | 56 | 57±9.6 | 0 | NA |  |
|  |  |  |  |  |  |  |  |  |  |
|  |  |  |  |  |  |  |  |  |  |
| Eisen 1993^[27]^ | Lamotrigine | 100mg | Canada | 18 | 34 | 57.5±13.0 | 0 | NA | Death |
|  | Placebo |  |  |  | 33 | 58.6±10.7 | 0 | NA |  |
|  |  |  |  |  |  |  |  |  |  |
| Rpghi 1993^[28]^ | BCAA | 24g | Italy | 12 | 61 | 59.8±10.9 | 0 | NA | Death |
|  | Placebo | - |  |  | 65 | 57.3±10.5 | 0 | NA |  |
|  |  |  |  |  |  |  |  |  |  |
| Cudkowicz 2013^[29]*^ | Dexpramipexole | 300mg | International | 9 | 474 | 56.8±11.3 | 76 | 38.4 | Death |
|  | Placebo |  |  |  | 468 | 57.3±11.3 | 76 | 37.9 |  |
|  |  |  |  |  |  |  |  |  |  |
| Beghi 2013^[30]*^ | Acetyl-L-carnitine | 3000mg | Italy | 12 | 157 | NA | 73.8 | 26.4 | Death/tracheostomy |
|  | Placebo | - |  |  | 153 | NA | 74 | 25.3 |  |
|  |  |  |  |  |  |  |  |  |  |

| **ALSFRS-R** | Intervention | Dose | Location | Duration,  month | | Sample  size | | Age,  year | Use of  rizuloze% | | Baseline of  ALSFRS-R |
| --- | --- | --- | --- | --- | --- | --- | --- | --- | --- | --- | --- |
| Dalla 2021^[31]^ | Guanabenz | 16-64mg | Italy | 6 | | 151 | | 59.3±11.3 | 93.3 | | 37.7 |
|  | Placebo | - |  |  | | 49 | | 61±12 | 98 | | 38 |
|  |  |  |  |  | |  | |  |  | |  |
| Kaji 2019^[32]^ | Methylcobalamin | 25-50mg | Japan | 3 | | 247 | | 61.6±9.85 | 89.85 | | 39.85 |
|  | Placebo | - |  |  | | 123 | | 62.2±10.7 | 89.4 | | 40.1 |
|  |  |  |  |  | |  | |  |  | |  |
| Rubia 2019^[33]^ | EH301 | 10mg | Spain | 4 | | 13 | | 56.9±9.1 | 100 | | 38.8 |
|  | Placebo | - |  |  | | 14 | | 55.6±10.5 | 100 | | 41.5 |
|  |  |  |  |  | |  | |  |  | |  |
| Koji 2017^[34]^ | Edaravone | 10mg | Japan | 6 | | 69 | | 60.5±10 | 91 | | 41.9 |
|  | Placebo | - |  |  | | 68 | | 60.1±10 | 91 | | 41.8 |
|  |  |  |  |  | |  | |  |  | |  |
| Amirzagar 2015^[35]^ | G-CSF | 0.01mg/kg | Iran | 3 | | 20 | | 51.3±8.6 | 70 | | 33.3 |
|  | Placebo | - |  |  | | 20 | | 52.5±11.6 | 70 | | 36.6 |
|  |  |  |  |  | |  | |  |  | |  |
| Abe 2014^[36]^ | Edaravone | 60mg | Japan | 6 | | 101 | | NA | 72 | | NA. |
|  | Placebo |  |  |  | | 104 | | NA | 72 | | NA. |
|  |  |  |  |  | |  | |  |  | |  |
| Morrison 2013^[37]^ | Lithium | 295mg | UK | 18 | | 107 | | 59.7±9.9 | 100 | | 38.20 |
|  | Placebo |  |  |  | | 107 | | 59.5±11.5 | 100 | | 38.64 |
|  |  |  |  |  | |  | |  |  | |  |
| Cudkowicz 2011^[38]^ | Dexpramipexole | 50/150/300mg | USA | 3 | | 75 | | 57.4±10.73 | 61.4 | | 38.3 |
|  | Placebo | - |  |  | | 27 | | 55.8±9.07 | 59.3 | | 37.3 |
|  |  |  |  |  | |  | |  |  | |  |
| Pascuzzi 2010^[39]^ | Talampanel | 150mg | American | 9 | | 40 | | 56.3 | 60 | | 32.6 |
|  | placebo | - |  |  | | 19 | | 52.6 | 58 | | 32.8 |
| Nefussy 2010^[40]^ | G-CSF | 5mg/kg | UK | 12 | | 19 | | 56.9±2 | NA | | 36.1 |
|  | Placebo | - |  |  | | 20 | | 53.4±10 | NA | | 34.6 |
|  |  |  |  |  | |  | |  |  | |  |
| Aggarwal 2010^[41]^ | Lithium |  | N-America | 6 | | 40 | | 58.3±10.2 | 100 | | 38.4 |
|  | Placebo |  |  |  | | 44 | | 55.5±11.9 | 100 | | 36.5 |
|  |  |  |  |  | |  | |  |  | |  |
| Cudkowicz 2006^[42]^ | Celecoxib | 800mg | USA | 12 | | 201 | | 54.5±11.8 | 67 | | 42.88 |
|  | Placebo | - |  |  | | 99 | | 55.0±12.4 | 71 | | 43.24 |
|  |  |  |  |  | |  | |  |  | |  |
| Shefner 2004^[43]^ | Creatine | 5-20g | USA | 6 | | 50 | | 59±12.5 | 54 | | 42 |
|  | Placebo | - |  |  | | 54 | | 59±10.8 | 50 | | 41 |
|  |  |  |  |  | |  | |  |  | |  |
| Weiss 2016^[44]^ | Mexiletine | 900 mg | France | 4 | | 39 | | 57.5±8.6 | 66.7 | | 34.95 |
|  | Placebo |  |  |  | | 20 | | 59.2±7.1 | 75 | | 34.9 |
|  |  |  |  |  | |  | |  |  | |  |
| Saccà2012^[45]^ | IGF | 2-8IU | Italy | 12 | | 20 | | 63.7±8.5 | 100 | | 34.5 |
|  | Placebo |  |  |  | | 20 | | 61.7±8.3 | 100 | | 36.6 |
|  |  |  |  |  | |  | |  |  | |  |
| Kaufmann 2009^[46]^ | CoQ10 | 2700mg | USA | 9 | 75 | | 56.5±10.8 | | 76.0 | 35.3 | |
|  | Placebo |  |  |  | | 75 | | 57.4±11.0 | 66.7 | | 35.6 |
|  |  |  |  |  | |  | |  |  | |  |
| Vucic 2021^[^ ^47]^ | Dimethyl fumarate | 480mg | Australia | 9 | | 72 | | 60.1±9.8 | 81 | | 38.6 |
|  | Placebo | - |  |  | | 35 | | 58.7±11.0 | 77 | | 38.7 |
|  |  |  |  |  | |  | |  |  | |  |

Note: NA：no reported ; NIV: non-invasive ventilation; PAV: permanent assisted ventilation; *: This literature also reports ALSFRS-R and included in the analysis

**Table S4 Covariate screening for inclusion**

| **Covariates** | **OFV** | **△OFV** |
| --- | --- | --- |
| **Oerall survival model** |  |  |
| Base model | -2304.602 | - |
| Age of onset on Hazonw | -2288.511 | - |
| Duration on Hazonw | -2315.987 | **11.385>3.84** |
| Male on Hazonw | -2306.384 | 1.782 |
| Riluzole on Hazonw | -2310.725 | **6.123>3.84** |
| Bulbar on Hazonw | -2304.634 | 0.032 |
| ALSFRS on Hazonw | -2304.653 | 0.051 |
|  |  |  |
| **ALSFRS-R model** |  |  |
| Base model | 35.081 | - |
| Bulbar on E_max_ | 34.451 | 0.63 |
| ALSFRS on E_max_ | 34.260 | 0.821 |

*OFV, Objective function value; HAZONW, cumulative risk of death in OS model; E_max_, the maximum effect in ALSFRS-R model

Final model: $h\left( t \right)=h(t){\_}_{Base}*e^{\left( DURATION-17.8 \right)*0.0317-RILUZOLE*0.228}$

(a)


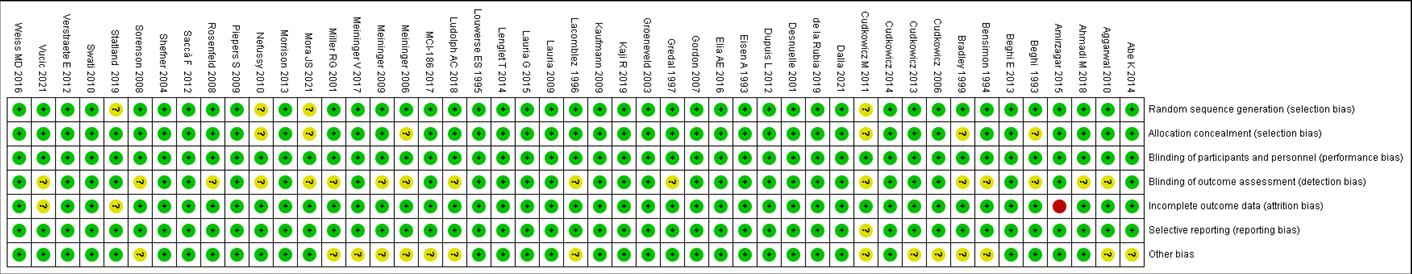


(b)


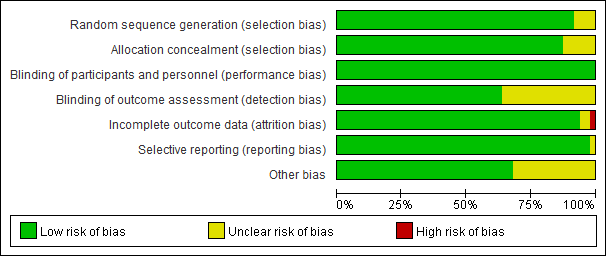


**Figure S1 Risk of bias assessment chart**.

Note: Using Cochrane’s risk of bias assessment tool to assess the study-level (a) and overall (b) risk of bias. In this tool, studies were deemed to be at high, low or unclear risk of bias based on adequacy of sequence generation, allocation concealment, blinding, processing of incomplete data, selective reporting, and other biases

(a)


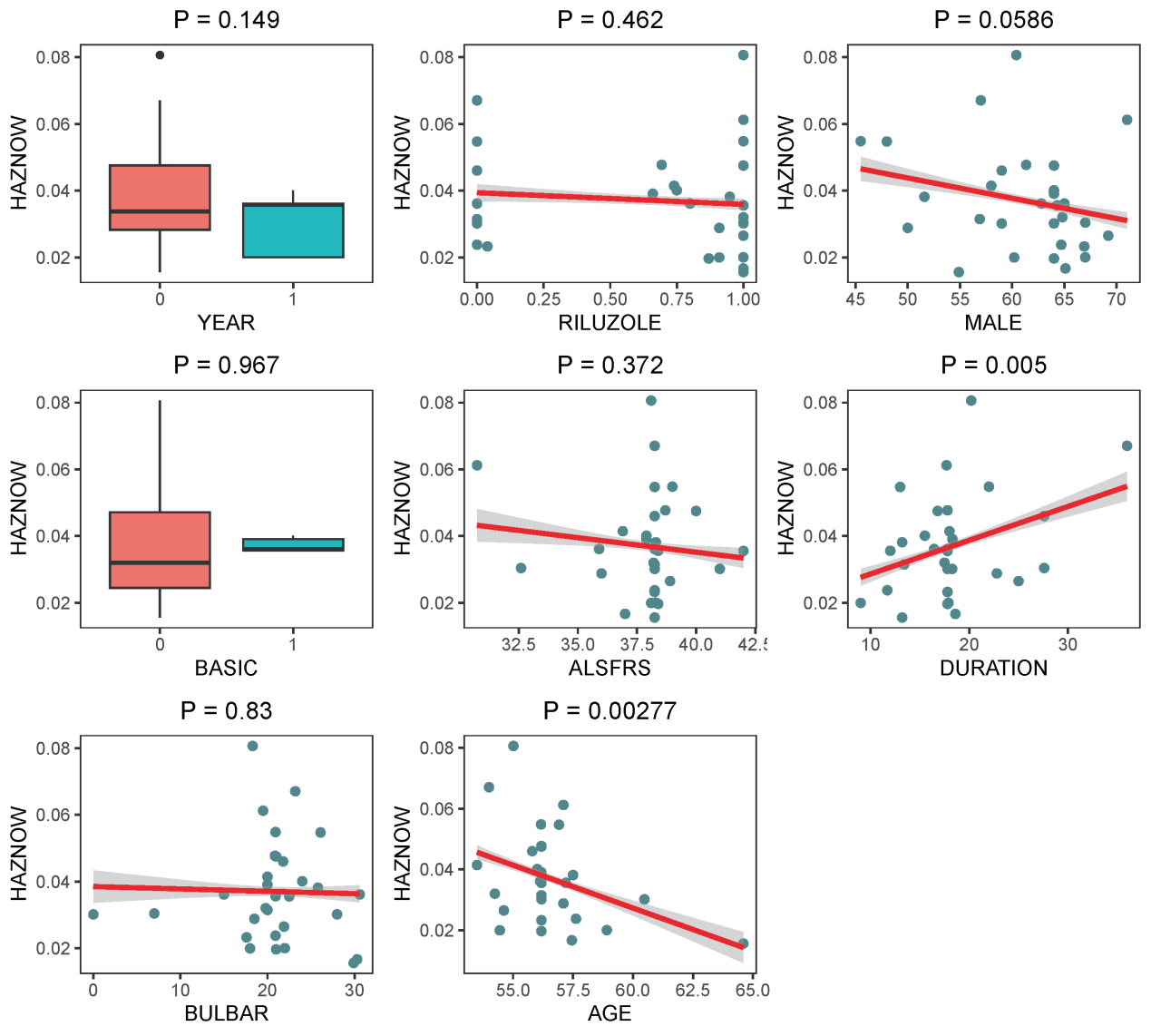


(b)


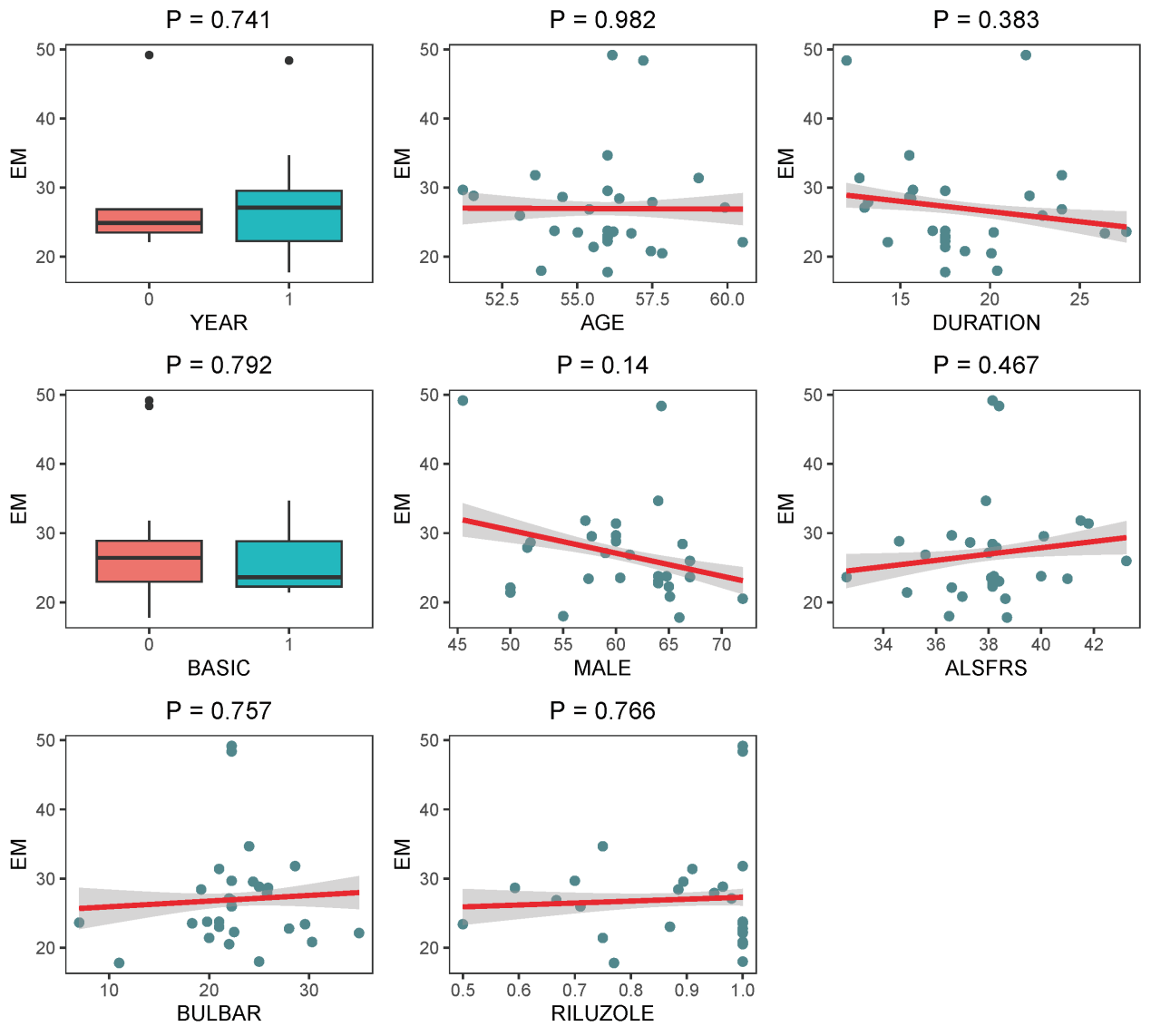


**Figure S2 Correlation analysis between covariates and model parameters**

Note: HAZONW, cumulative risk of death within 29 months in OS model; EM, the maximum effect in ALSFRS-R model; In the sub-graph of BASIC, 1 represents subjects with basic therapy, and 0 represents subjects with no basic therapy; In the sub-graph of YEAR, 1 represents the studies were published after 2009, and 0 represents the studies were published before 2009; In the sub-graph of BULBAR, the value of the abscissa represents the proportion of individuals with bulbar onset; In the sub-graph of MALE, the value of the abscissa represents the proportion of male; In the sub-graph of DURATION, the value of the abscissa represents the time from symptom onset to inclusion; In the sub-graph of AGE, the value of the abscissa represents the age of the patients; In the sub-graph of RILUZOLE, the value of the abscissa represents the proportion of patients receiving riluzole; In the sub-graph of ALSFRS-R, the value of the abscissa represents the ALSFRS-R score in baseline; In scatter plots, the solid line represents the trend line and the shaded area represents the 95% CI of the trend line.

(a)


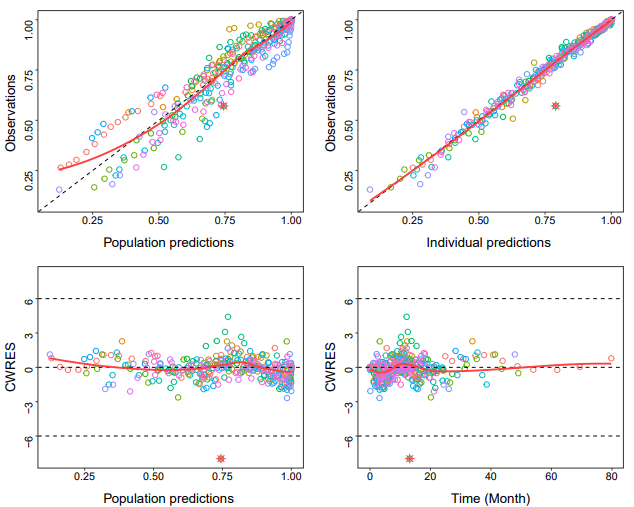


(b)


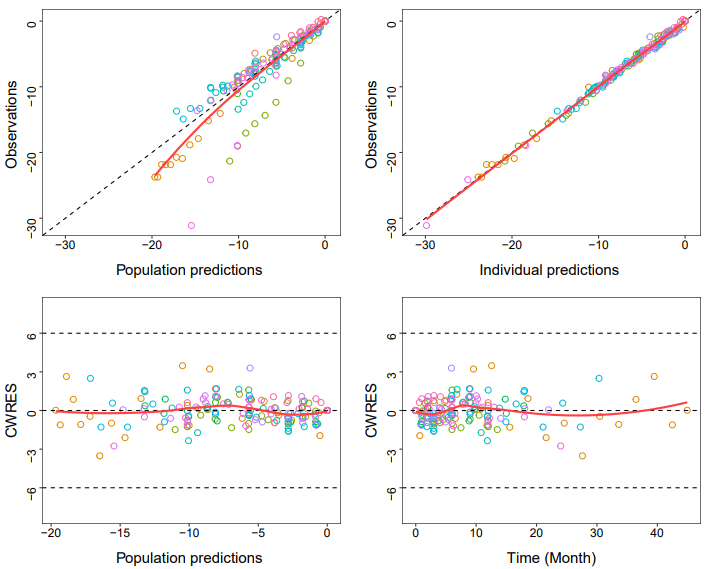


**Figure S3 The goodness-of-fit plots of the final model of OS and ALSFRS-R**

Note: (a): OS model; (b)ALSFRS-R model; The upper left, population predictions versus observations. The upper right, individual predictions versus observations. The lower left, conditional weighted residuals (CWRES) versus population predictions. The lower right, conditional weighted residuals (CWRES) versus time. The dashed black lines in the upper left and right panels are diagonal, and the dashed black lines in the lower left and right panels are the 0 line and the ± 6 reference line, respectively.

The model diagnostic plots showed that the observations (OBS), population predictions (PRED), OBS, and individual predictions (IPRED) of the final OS and ALSFRS-R models were evenly distributed on both sides of the diagonal line, with the fitted lines coinciding with the diagonal line. The conditional weighted residual errors (CWRES) of most points were distributed evenly around the 0 line within 6, and the fitting lines of CWRES vs. PRED and CWRES vs. time nearly coincided with the 0 line


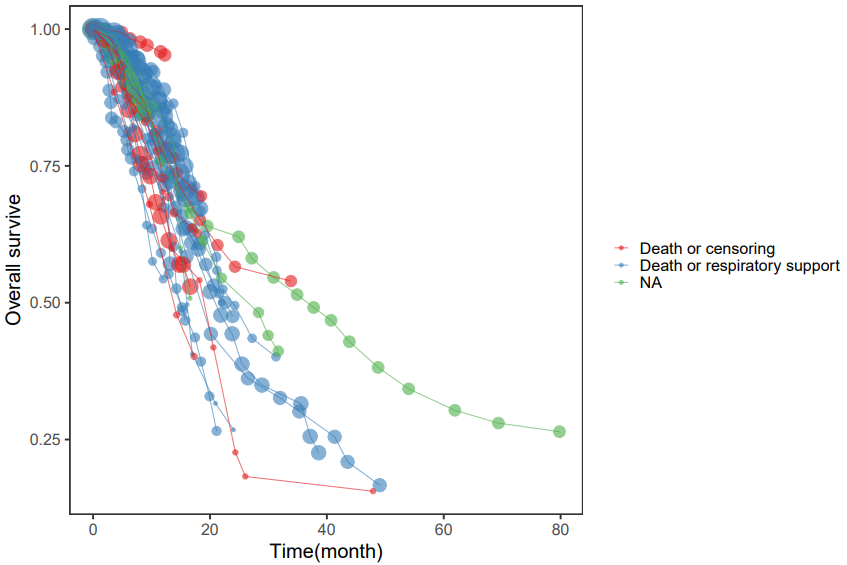


**Figure S4 Overall survival data view with three different death definition.**

Note: three different colors represent three definitions of death, namely: death or censoring, death or respiratory support and NA (no specific definition of death); The size of points is related to the sample size.
